# Neuroepithelial control of mucosal inflammation in acute cystitis

**DOI:** 10.1038/s41598-018-28634-0

**Published:** 2018-07-20

**Authors:** Daniel S. C. Butler, Ines Ambite, Karoly Nagy, Caterina Cafaro, Abdulla Ahmed, Aftab Nadeem, Nina Filenko, Thi Hien Tran, Karl-Erik Andersson, Björn Wullt, Manoj Puthia, Catharina Svanborg

**Affiliations:** 1 Department of Microbiology, Immunology and Glycobiology, Institute of Laboratory Medicine, Lund University, 223 62 Lund, Sweden; 2 Jahn Ferenc (South Pest) Teaching Hospital, 1204 Budapest, Hungary; 3 Institute for Regenerative Medicine, Wake Forest University School of Medicine, Winston Salem, NC USA; 4 Institute of Clinical Medicine, Department of Obstetrics and Gynecology, Aarhus University, 8200 Aarhus, Denmark

## Abstract

The nervous system is engaged by infection, indirectly through inflammatory cascades or directly, by bacterial attack on nerve cells. Here we identify a neuro-epithelial activation loop that participates in the control of mucosal inflammation and pain in acute cystitis. We show that infection activates Neurokinin-1 receptor (NK1R) and Substance P (SP) expression in nerve cells and bladder epithelial cells *in vitro* and *in vivo* in the urinary bladder mucosa. Specific innate immune response genes regulated this mucosal response, and single gene deletions resulted either in protection (*Tlr4*^−/−^ and *Il1b*^−/−^ mice) or in accentuated bladder pathology (*Asc*^−/−^ and *Nlrp3*^−/−^ mice), compared to controls. NK1R/SP expression was lower in *Tlr4*^−/−^ and *Il1b*^−/−^ mice than in C56BL/6WT controls but in *Asc*^−/−^ and *Nlrp3*^−/−^ mice, NK1R over-activation accompanied the exaggerated disease phenotype, due, in part to transcriptional de-repression of *Tacr1*. Pharmacologic NK1R inhibitors attenuated acute cystitis in susceptible mice, supporting a role in disease pathogenesis. Clinical relevance was suggested by elevated urine SP levels in patients with acute cystitis, compared to patients with asymptomatic bacteriuria identifying NK1R/SP as potential therapeutic targets. We propose that NK1R and SP influence the severity of acute cystitis through a neuro-epithelial activation loop that controls pain and mucosal inflammation.

## Introduction

Infections are accompanied by characteristic symptoms from the site of infection and by general malaise, in case of systemic involvement. Pain serves as a key indicator of disease severity and as a warning signal for the host. Pain is also one of the classical hallmarks of inflammation, together with hyperemia, edema and increased temperature in inflamed tissue foci. Neuropeptides and receptors that mediate nociception and pain signalling include Substance P (SP) and its receptor Neurokinin-1 receptor (NK1R)^1,2^. SP is secreted by nerves and inflammatory cells and affects nociceptive signalling in the dorsal horn and the dorsal root ganglia. SP mediates interactions between neurons and immune cells and nerve-derived SP modulates immune cell proliferation rates and cytokine production^3,4^. Interestingly, the nervous system senses the presence of microbes and participates actively in the antimicrobial defense. As virulence factors engage specific receptors on nerve cells, bacteria activate ion fluxes, leading to nerve cell activation^5^. Examples of such interactions include recognition of Lipopolysaccharide (LPS) by Toll like receptor-4 (TLR-4) and of Shiga toxin by glycolipid receptors^6,7^. Specific nerve cell activation products also modulate inflammation, suggesting broad relevance for a number of infection-induced disease states^8,9^.

Pain from the site of infection characterizes acute cystitis; an extremely common bacterial infection affecting about 50% of all women at least once^10^. In addition to painful urination (dysuria), characteristic clinical symptoms include urgency and frequency of urination, caused by activation of the micturition reflex and contractions of the bladder detrusor muscle^11^. This symptom profile indicates that the nervous system is engaged in the pathogenesis of acute cystitis^12^ but molecular determinants of this process have not been defined. NK1R and SP are activated in patients with interstitial cystitis and in pelvic pain models where pseudorabies virus and LPS O-antigen have been proposed as pain agonists^13–17^. The extent of nerve cell activation by pathogenic bacteria is not well understood, yet some studies on bacterial toxin and sensory nerve cell activation suggest an important TLR4-independent link between LPS and transient receptor potential channel A1, as well as *E. coli* derived formyl peptides and formyl peptide receptor 1^5,18^. However, the nerve cell determinants of acute cystitis have not been defined.

Recent immuno-genetic studies have identified a molecular basis for acute cystitis, involving Interleukin-1β (IL-1β) as a key regulator of the innate immune response to bladder infection. Uropathogenic *E. coli* activate IL-1β through a TLR4-dependent signaling pathway and as a result the acute cystitis phenotype is attenuated in *Tlr4*^−/−^ and *Il1b*^−/−^ mice^19,20^. *Asc*^−/−^ and *Nlrp3*^−/−^ mice, in contrast, develop severe acute cystitis, due to an IL-1β hyper-activation disorder^20^. In this study, we address if acute cystitis strains activate a neuropeptide- and neuropeptide receptor response in the urinary bladder mucosa and if the genes that regulate acute cystitis severity also control nerve cell activation. The study was prompted by the characteristic symptoms of acute cystitis and by preliminary evidence of neuropeptide receptor activation in patients with bacteriuria.

## Results

### Neuro-epithelial response to *E. coli* infection

**Figure 1:**
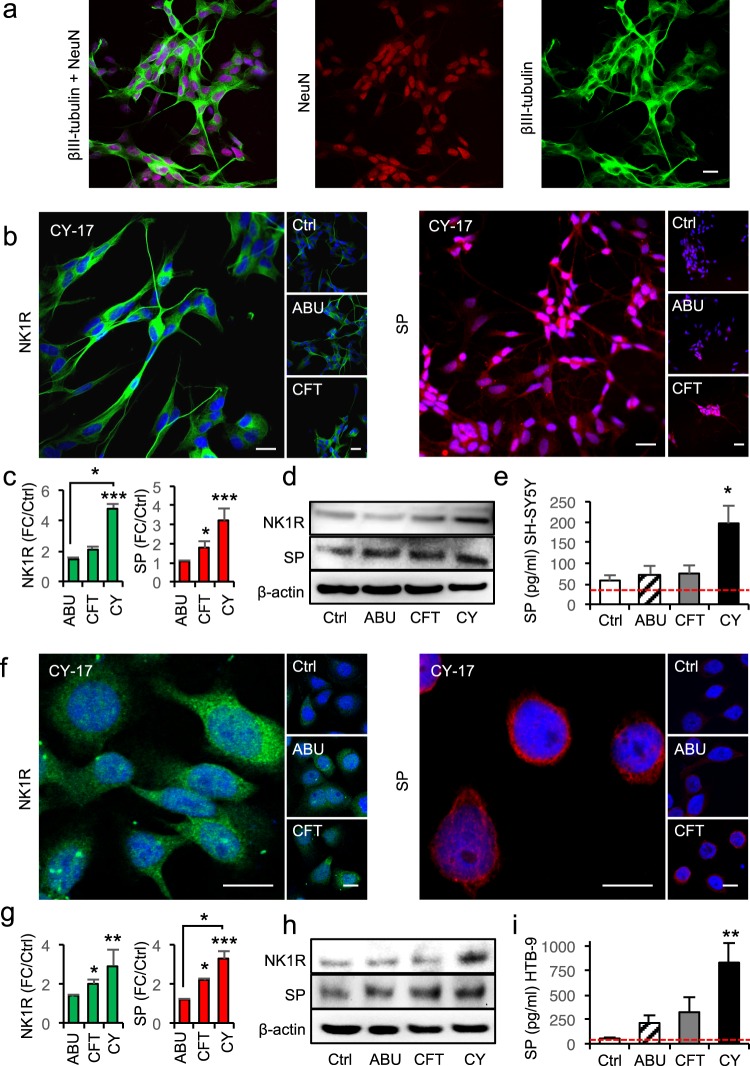
Bacterial SP/NK1R activation in differentiated nerve cells and bladder epithelial cells. (**a**) SH-SY5Y cells, differentiated using retinoic acid and serum starvation, showed characteristic morphology and staining for neuron-specific markers βIII-tubulin (green) and NeuN (red). (**b**) Increase in NK1R (green) and SP (red) staining after infection of differentiated nerve cells with the cystitis isolate CY-17, the APN strain CFT073 or the ABU strain *E. coli* 83972 (10^4^ CFU/ml, four hours). (**c**) Quantification of fluorescence intensities in (**b**). FC compared to uninfected cells (*n* = 50 cells per condition, four repeats) (**d**) Western blot confirming the increase in NK1R and SP protein levels in infected nerve cells (4 repeats). (**e**) Quantification of SP in supernatants from infected nerve cells compared to uninfected cells by ELISA, red line represents the detection limit of the ELISA (*n* = 7 samples, 2 repeats). (**f**) Increase in NK1R (green) and SP (red) staining in bladder epithelial cells infected with CY, CFT or ABU. (**g**) Quantification of fluorescence intensities in (**f**), FC compared to uninfected cells, (*n* = 50 cells per condition, four repeats). (**h**) Western blots confirming the increase in NK1R and SP protein levels in infected bladder epithelial cells (4 repeats). (**i**) SP levels in supernatants from bladder epithelial cells infected with ABU, APN or CY compared to uninfected cells, red line represents the detection limit of the ELISA (*n* = 6 samples, 2 repeats). The data is presented as means + SEMs and analysed using Kruskal Wallis test, Dunn’s correction. Scale bars = 20 µm. *P < 0.05, **P < 0.01, ***P < 0.001.

To address if infection activates neurokinin-1 receptor (NK1R) and its ligand Substance P (SP) in the urinary tract, we first infected nerve cells (SH-SY5Y) and bladder epithelial cells (HTB9) with relevant *E. coli* strains *in vitro* (Fig. 1). Prior to infection, the SH-SY5Y nerve cells were differentiated by treatment with Retinoic Acid (1%) and serum starvation for 7 days and differentiation was confirmed by staining for the neuronal markers βIII-tubulin and NeuN (Fig. 1a). The acute cystitis strain CY-17 was selected for these studies, based on its ability to stimulate IL-1β production and to induce bladder pathology *in vivo*, in the murine acute cystitis model^20^.

CY-17 infection stimulated dose-dependent cellular NK1R and SP responses, quantified by confocal imaging and western blot analysis (Fig. 1, Supplementary Fig. S1). Nerve cells and bladder epithelial cells showed similar dose response profiles, peaking at 10^4^ CFU/ml (MOI of 0.05). In addition, CY-17 stimulated the secretion of SP into cell supernatants (*P* < 0.05), (Fig. 1b–i). The NK1R and SP response to CY-17 infection was further investigated in two additional cell lines (MOI = 0.05, 4 hours). The DLD1 colonic epithelial cell line showed the same response kinetics as the SH-SY5Y and HTB9 cells. In infected kidney cells, in contrast, NK1R expression decreased compared to uninfected cells and SP expression was not affected (Supplementary Fig. S1).

We subsequently compared CY-17 to the acute pyelonephritis (APN) strain *E. coli* CFT073 and the asymptomatic bacteriuria (ABU) strain *E. coli* 83972 (MOI = 0.05, 4 hours, Fig. 1). The APN strain actively induced SP expression in nerve cells and NK1R and SP expression in bladder epithelial cells (*P* < 0.05 compared to uninfected cells). The ABU strain, in contrast, did not induce a SP or NK1R response. To further address if acute cystitis strains are efficient NK1R/SP inducers, we compared additional pediatric acute cystitis strains (*n* = 7) to pediatric ABU strains (*n* = 7)^21^. The acute cystitis strains activated NK1R and SP expression more efficiently than the ABU strains (*P* < 0.05, Supplementary Fig. S2).

### Neuro-epithelial response to bladder infection, *in vivo*

**Figure 2:**
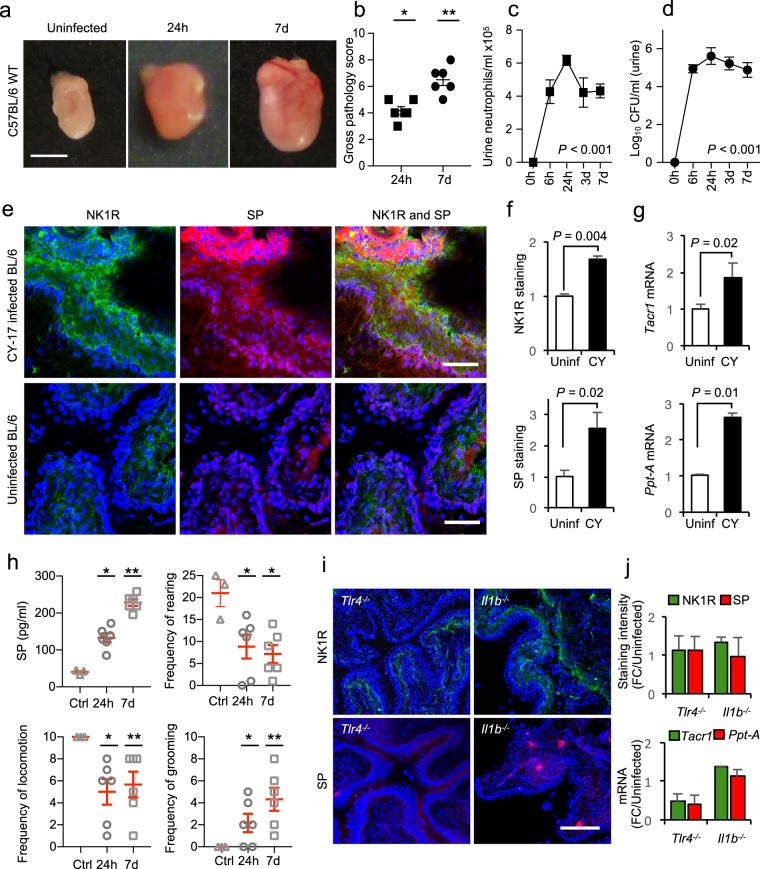
Mucosal neuropeptide response to acute cystitis. (**a**) Evidence of acute cystitis in C57BL/6WT mice infected with CY-17, defined by macroscopic inspection (Scale bar = 1 mm). (**b**) Gross pathology score of bladders from infected C57BL/6WT mice compared to uninfected controls. (**c**) Kinetics of the neutrophil response and (**d**) bacterial persistence, quantified in urine samples obtained 6 hours and 24 hours, 3 days and 7 days post infection. (**e**) NK1R and SP staining, quantified by immunohistochemistry of bladder sections. Mice were infected with CY-17 for 7 days. SP staining (red) was increased in the epithelium and NK1R (green) in the subepithelial compartment, compared to uninfected controls. (**f**) Quantification of NK1R and SP staining in (**e**). (**g**) Increased expression of *Tacr1* and *Ppt-A* in bladders infected with CY-17 for 7 days, quantified by qRT-PCR. (**h**) Urine concentration of SP detected by ELISA and pain assessed in C57BL/6WT mice after 24 hours and 7 days of CY-17 infection. Mice behavior was recorded and scored according to locomotion, frequency of rearing and frequency of grooming compared to uninfected controls. (**i**) Genes controlling the NK1R and SP response were identified by CY-17 infection of mice carrying single gene deletions known to affect the susceptibility to acute cystitis (*Tlr4*^−/−^*, Il1b*^−/−^ mice). NK1R (green) and SP (red) staining in infected *Tlr4*^−/−^ and *Il1b*^−/−^ mice. (**J**) Quantification of SP and NK1R staining in *Il1b*^−/−^ and *Tlr4*^−/−^ mice compared to their respective uninfected controls. Data is represented as means ± SEMs from *n* = 4–6 mice per group, (two repeats) and analysed by Mann-Whitney U-test. *P < 0.05 **P < 0.01, ***P < 0.001.

To address the *in vivo* relevance of these findings, we established acute cystitis in C57BL/6WT mice (Fig. 2)^20^. The severity of acute cystitis was quantified as the gross pathology score (edema, hyperemia and bladder enlargement). Neuropeptide expression was quantified by immunohistochemistry after staining with specific antibodies and by qRT-PCR of whole bladder RNA extracts, using primers specific for *Tacr1 and Ppt-A*.

In mice infected with CY-17, bladder pathology was detected after 24 hours and at seven days, compared to uninfected mice (*P* < 0.05, both time points Fig. 2a,b). Urine neutrophil counts increased after 6 hours and remained elevated until sacrifice on day 7 at 4–6 × 10^5^ cells per ml. Bacterial counts in urine showed similar kinetics and plateaued at 10^5^ CFU/ml (*P* < 0.001, Fig. 2c,d).

Infection stimulated an increase in mucosal NK1R and SP staining (*P* = 0.004 and *P* = 0.02 compared to uninfected controls, Fig. 2e,f). NK1R was clearly visible in infected bladders, with a distinct staining pattern of the mucosal nerve plexus in the lamina propria. Using βIII tubulin as a neuronal marker, co-localization of nerve cells with NK1R was confirmed. SP, in contrast, was mainly observed in the epithelial layer of infected bladders. Co-localization with βIII tubulin was more restricted than for NK1R and only detected along the epithelial-nerve cell interface (Supplementary Fig. S3).

The increase in NK1R and SP expression was confirmed by qRT-PCR of total bladder RNA (Fig. 2g). *Tacr1* and *Ppt-A* mRNA levels were increased, compared to uninfected mice (*P* = 0.02 and *P* = 0.008 for *Tacr1* and *Ppt-A* respectively). Urine SP levels were elevated after 24 hours and 7 days in infected C57BL/6WT mice compared to uninfected controls (128 pg/ml and 217 pg/ml respectively, compared to 43 pg/ml, *P* < 0.05, Fig. 2H).

Symptoms were documented by video recording of the mice before and at defined times post infection (24 hours and 7 days). A significant change in behavior was detected and quantified as a decrease in rearing and locomotion and an increase in grooming behavior (*P* < 0.05 for each of the three variables compared to uninfected controls, Fig. 2h). The results suggest that acute cystitis in C57BL/6WT mice is accompanied by a mucosal neuropeptide response and symptoms from the site of infection.

### NK1R and SP response *in Tlr4*^−/−^ and *IL1b*^−/−^ mice

*Tlr4* is an essential, upstream regulator of the host response to gram-negative bacterial infection^22^. Downstream signaling varies with the virulence repertoire of the infecting strain and in acute cystitis, IL-1β is critically involved in the generation of tissue pathology^20^. To examine if TLR-4 and IL-1β also regulate the neuropeptide response, we infected *Tlr4*^−/−^ and *Il1b*^−/−^ mice with the CY-17 strain and compared NK1R and SP staining to C57BL/6WT mice with intact TLR-4 and IL-1β function.

NK1R or SP levels were low in bladder tissue from infected *Tlr4*^−/−^ and *Il1b*^−/−^ mice, with little change after 7 days (Fig. 2i,j, Supplementary Fig. S4). Significant bladder pathology was not detected in these mice. The results identify *Tlr4* and *Il1b* as important upstream regulators of mucosal NK1R and SP responses.

### NK1R and SP hyper-activation in *Nlrp3*^−/−^ and *Asc*^−/−^ mice

The inflammasome proteins NLRP3 and ASC control the processing of pro-IL-1β together with caspase-1. In previous studies, we have identified a new, non-canonical mechanism of IL-1β processing, involving the metalloproteinase MMP7. This pathway is hyperactive in *Nlrp3*^−/−^ and *Asc*^−/−^ mice, which develop IL-1β driven hyper-inflammation and severe acute cystitis^20^.

**Figure 3:**
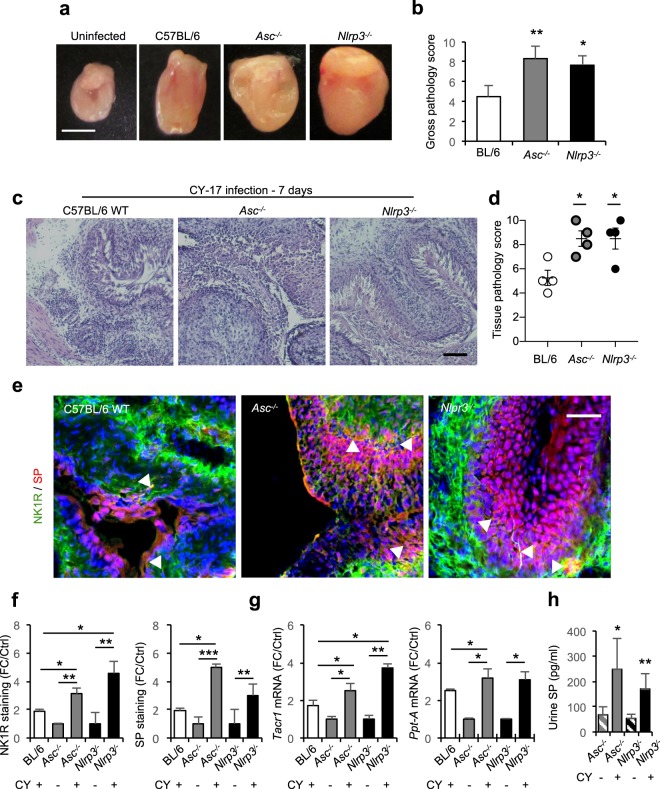
Severe acute cystitis is accompanied by a strong mucosal neuropeptide response in *Asc*^−/−^ *and Nlrp3*^−/−^ mice. (**a**) Severe acute cystitis in *Asc*^−/−^ *and Nlrp3*^−/−^ mice infected with CY-17 compared to uninfected mice, defined by macroscopic inspection (7 days post infection). (**b**) Gross pathology score of infected bladders. (**c**) Tissue pathology, defined by H&E staining of frozen bladder sections. Increase in inflammatory cell infiltration, edema and loss of tissue structure definition. (**d**) Increased tissue pathology score in infected *Asc*^−/−^ *and Nlrp3*^−/−^ mice, defined by histology. (**e**) Augmented NK1R (green) and SP (red) staining in infected *Asc*^−/−^ *and Nlrp3*^−/−^ mice bladder tissue. White arrowheads indicate points of co-localization. (**f**) Quantification of NK1R and SP staining in (**e**). (**g**) Elevated *Tacr1* and *Ppt-A* mRNA levels in *Asc*^−/−^ and *Nlrp3*^−/−^ mice, normalized against uninfected controls. (**h**) Elevated urine SP levels in *Asc*^−/−^ and *Nlrp3*^−/−^ mice infected with CY-17. Data is presented as means ± SEMs from *n* = 3–5 mice and analysed using Kruskal Wallis test with Dunn’s correction. The response kinetics was analysed by AUC *Welsch’s t-*test. *P < 0.05, **P < 0.01, ***P < 0.001.

To examine if the NK1R/SP response is controlled by a similar mechanism, *Asc*^−/−^ and *Nlrp3*^−/−^ mice were infected with CY-17 and sacrificed 7 days post infection. The gross pathology score was higher in *Asc*^−/−^ and *Nlrp3*^−/−^ mice than in C57BL/6WT mice, as determined by enlarged bladders, edema and hyperemia (*P* = 0.008 and *P* = 0.034 respectively, Fig. 3a,b). The disease response was further accompanied by general tissue destruction, as shown by a massive neutrophil influx and epithelial hyperplasia, compared to uninfected controls (Fig. 3c,d).

Bladder pathology was accompanied by an increase in NK1R and SP staining in infected *Asc*^−/−^ and *Nlrp3*^−/−^ mice (*P* < 0.01 compared to uninfected controls, Fig. 3e,f). SP was detected throughout the epithelial layer but NK1R staining mainly in the lamina propria with co-localization basolaterally, along the interphase between the nerve plexus and the epithelium. NK1R-positive fibers between the epithelial cells were observed in inflamed regions with epithelial hyperplasia. Further, *Tacr1* and *Ppt-A* expression was higher in *Asc*^−/−^ and *Nlrp3*^−/−^ mice than in C57BL/6WT mice (*P* < 0.05, Fig. 3g) and urine SP levels were elevated compared to uninfected controls (248 pg/ml and 170 pg/ml respectively, *P* < 0.05, Fig. 3h). The results suggest that *Asc* and *Nlrp3* regulate important aspects of NK1R and SP expression in acute cystitis.

### Contributions of neutrophils and macrophages

To address if recruited neutrophils or resident macrophages express NK1R and SP, tissue sections from infected C57BL/6WT- or *Nlrp3*^−/−^ mice were stained for NK1R- and SP and counter-stained with neutrophil- or macrophage-specific antibodies. While a massive neutrophil influx was detected, there was little evidence of co-localization with NK1R or SP in most infiltrating neutrophils. Scattered sub-epithelial macrophages were visible in infected mice, but showed no staining for NK1R or SP (Supplementary Fig. S3). The results suggest that resident nerve and epithelial cells are important sources of NK1R and SP, also in the hyper-inflamed mucosa.

### Effects of NK1R inhibition on mucosal inflammation

**Figure 4:**
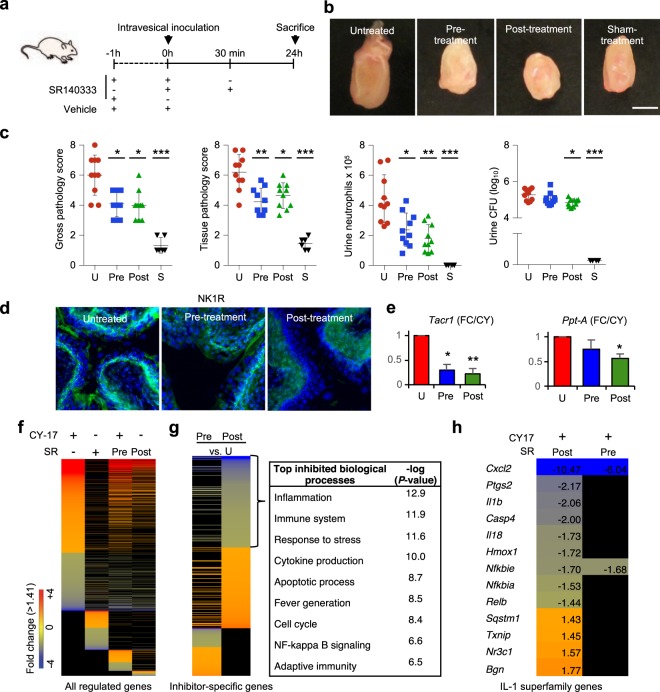
Therapeutic effect of NK1R inhibition in *Nlrp3*^−/−^. (**a**) Treatment protocol. The irreversible, non-peptide NK1R antagonist SR140333 was used to inhibit the NK1R-dependent host response to CY-17 infection. Susceptible *Nlrp3*^−/−^ mice were either pre-treated with SR140333 or treated post-infection (1 mg/kg i.p. 30 minutes prior to, or 1 hour after infection) before being sacrificed after 24 hours. Infected, un-treated mice were used as controls. (**b**) Protective effect of SR140333 treatment, shown by macroscopic inspection of infected bladders. Scale bar = 1 mm. (**c**) Decrease by SR140333 treatment of gross bladder pathology score, tissue pathology score (defined by H&E staining) and urine neutrophil counts. No change significant in bacterial counts in urine. (**d**) Inhibition of NK1R (green) staining in SR140333 treated *Nlrp3*^−/−^ mice compared to infected, untreated controls. Scale bar = 100 µm. (**e**) Reduced bladder *Tacr1* and *Ppt-A* expression in SR140333 treated *Nlrp3*^−/−^ mice by qRT-PCR (*n* = 5 mice. (**f**) Reduced gene expression in SR140333 treated mice compared to infected untreated controls defined by gene expression analysis of whole bladder mRNA (*n* = 2 mice per group, red = up-regulated, blue = down-regulated, *P* < 0.05, FC > 1.41, compared to uninfected controls). (**g**) Heat-map showing genes affected by SR treatment (*P* < 0.05, FC > 1.41 compared to infected untreated controls). Inhibited biological processes included inflammation and innate immune signaling. (**h**) Inhibition of inflammasome- and IL-1β related genes by SR140333 treatment, compared to untreated infected controls. Data is presented as means ± SEMs *n* = 10 mice per group (two repeats). Data was analyzed by Kruskal Wallis with Dunn’s correction, *P < 0.05, **P < 0.01, ***P < 0.001.

To inhibit the neuropeptide response *in vivo*, we used the irreversible non-peptide NK1R antagonist SR140333, which prevents SP from binding NK1R and is suitable for *in vivo* use^23^*. Nlrp3*^−/−^ mice were given SR140333 or vehicle intra-peritoneally, one hour before infection or 30 minutes after infection with CY-17 (Fig. 4a). The severity of acute cystitis was quantified as the gross pathology score and tissue pathology score after 24 hours (Figs 4b,c and S5). SR140333 reduced the gross pathology (*P* < 0.05 for pre-and post-infection treatment), tissue pathology (*P* = 0.005 and *P* = 0.03 for pre-and post-infection treatment) as well as urine neutrophil recruitment (*P* = 0.02 and *P* = 0.002 for pre-and post-infection treatment). The post-treatment also affected bacterial clearance, as shown by a reduction in urine CFUs (*P* = 0.02, Fig. 4c). SR140333 treatment inhibited NK1R staining in infected bladders and the reduction in NK1R expression was confirmed by qRT-PCR (Fig. 4d,e).

The effect of SR140333 treatment was subsequently confirmed in C57BL/6WT mice, using the post-infection treatment protocol (Supplementary Fig. S5). SR140333 treated mice showed a significant reduction in gross pathology (*P* = 0.02) and the tissue pathology score and urine neutrophil counts were reduced (*P* < 0.001 after 24 hours and *P* < 0.05 after 7 days, Supplementary Fig. S5).

The effects of SR140333 inhibition were validated, *in vitro*, using two additional NK1R antagonists (CP99994 and L703.606). Pre-treatment of bladder epithelial cells (30 min) reduced the NK1R and SP response to CY-17 infection, to the same extent as SR140333 (Supplementary Fig. S6, MOI = 0.05, 4 hours). In addition, a dose-dependent effect on cellular ATP levels was detected, consistent with the known mechanism of action of SR140333 (Supplementary Fig. S6). The results identify NK1R as a potential therapeutic target in acute cystitis.

### Inhibition of IL-1β-dependent mucosal inflammation by SR140333

To further understand the protective effect of NK1R inhibition, we analyzed the profile of genes expressed in SR140333-treated *Nlrp3*^−/−^ mice (Fig. 4f). About 50% of regulated genes were suppressed by SR140333, including genes involved in sensory perception of pain (Supplementary Fig. S7). Furthermore, SR140333 reduced the expression of inflammasome- and IL-1-superfamily genes by about 70%, including *Il18, Il33, Il6* and *ll1b* (Fig. 4g,h and Supplementary Table S1). *Cxcl2*, which encodes the neutrophil chemoattractant MIP-2/Groβ was the most strongly inhibited gene, consistent with the reduced number of neutrophils in treated mice, compared to untreated controls.

### Inhibition of NK1R/SP responses by Anakinra

**Figure 5:**
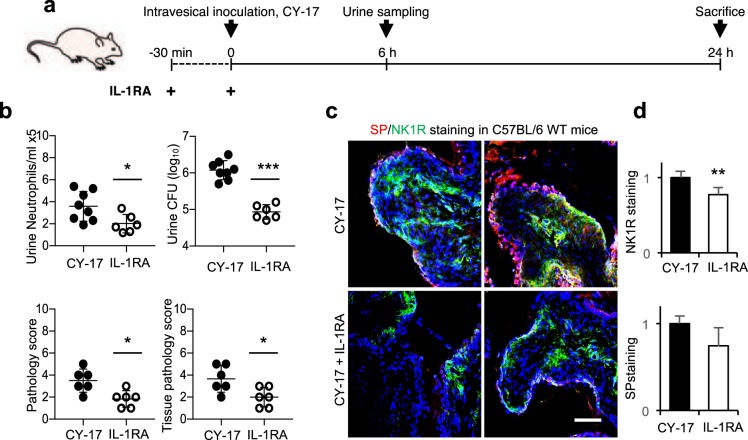
The IL-1 receptor antagonist Anakinra reduces mucosal neurokinin response. (**a**) Treatment protocol. C57BL/6WT mice were treated with the IL-1R antagonist Anakinra (IL-1RA), 30 minutes prior to infection and at the time of infection (1 mg/kg) with CY-17 and were sacrificed after 24 hours (*n* = 6 from two experiments). (**b**) IL-1RA treatment decreased the gross bladder pathology score, tissue pathology score (defined by H&E staining) and urine neutrophil counts as well as bacterial counts in urine compared to untreated, infected controls. (**c**) Reduction in NK1R (green) and SP (red) staining in IL-1RA treated mice compared to untreated, infected controls. Data from two representative mice are shown. (**d**) Quantification of NK1R and SP staining in (**c**). Data is represented as means ± SEMs and was analyzed by Mann-Whitney U-test, *P < 0.05, **P < 0.01, ***P < 0.001.

The attenuation of the NK1R/SP response in *Il1b*^−/−^ mice and the effects of the NK1R inhibitor on the expression of IL-1-superfamily genes identified IL-1β as a potential regulator of the neuropeptide response. To address this question, we used the IL-1 receptor (IL-1R) antagonist Anakinra^®^, which has shown therapeutic activity against acute cystitis in *Asc*^−/−^ mice^20^. Anakinra^®^ pre-treatment reduced SP/NK1R expression in C57BL/6 mice compared to sham treated controls, suggesting that IL-1β regulates neuropeptide levels in the infected bladder mucosa (*P* = 0.04, Fig. 5a–d). Furthermore, we observed a reduction in acute bladder pathology in treated mice, as well as bacterial and neutrophil counts in urine (24 hours *P* < 0.05, Fig. 5b).

This mutually inhibitory effect suggests that an activation loop involving NK1R, SP and IL-1β controls the inflammatory response in infected bladders.

### Transcriptional regulation of NK1R and SP expression

**Figure 6:**
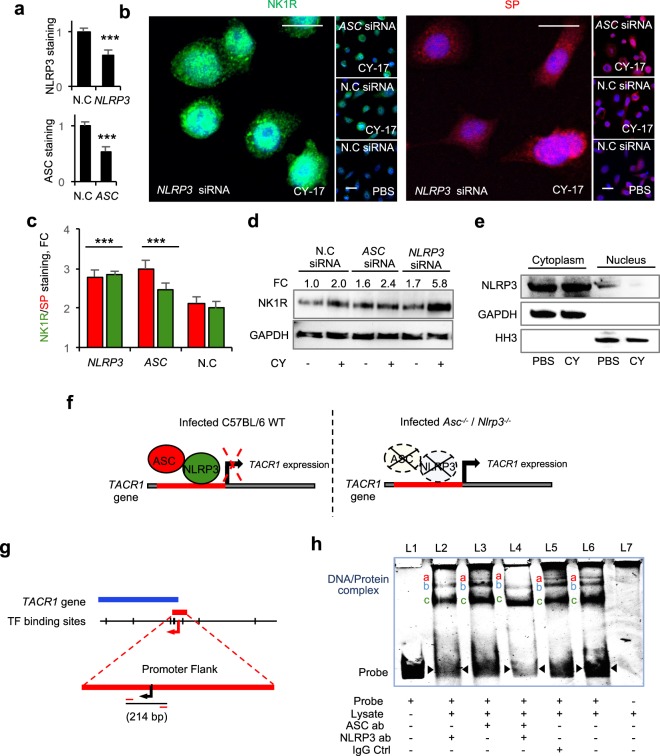
Transcriptional regulation of NK1R by ASC and NLRP3. To understand if NLRP-3 and ASC regulate the mucosal neurokinin response, bladder epithelial cells were transfected with specific siRNA prior to CY-17 infection and NK1R expression was quantified by confocal imaging and western blot analysis of whole cell lysates. (**a**) Evaluation of the efficacy of the siRNA treatments to inhibit ASC and NLRP3 expression in cells transfected with *ASC* or *NLRP3* siRNA (quantification of immunocytochemistry staining, FC to N.C.). (**b**) Confocal imaging of the siRNA effects on NK1R (green) and SP (red) response to infection in siRNA transfected bladder epithelial cells compared to scrambled, negative control siRNA (N.C). (**c**) Quantification of staining intensity of CY-17 infected cells in (**b**), (FC to uninfected negative control siRNA, means + SEMs (*n* = 3 experiments, 50 cells per condition). (**d**) Western blot analysis of whole cell lysates confirming the increase in NK1R seen in (**b**) (FC to uninfected negative control siRNA, *n* = 3 blots). (**e**) NLRP-3 levels in cytoplasmic and nuclear fractions of bladder cells with or without infection with CY-17 as shown by Western blot analysis (*n* = 2 blots). (**f**) Tentative model depicting transcriptional control of *TACR1* expression by ASC and NLRP3. The model predicts that the ASC/NLRP3 complex represses *TACR1* expression by binding to the *TACR1* promoter. *TACR1* expression is therefore de-repressed in cells lacking *ASC* or *NLRP3*, leading to increased cellular NK1R levels. (**g**) Representation of the *TACR1* gene with promoter flank showing the 214 bp DNA fragment used for of electrophoretic mobility shift assay (EMSA). (**h**) DNA band shifts were detected in the presence of whole cell extracts (bands a, b and c in lane 6). Specificity for NLRP-3, shown by competition with NLRP-3-specific antibodies (lane 2). Band (**a**) was removed and band (**b**) was attenuated. The ASC-specific antibody had no independent effect (lane 3), but the NLRP-3-and ASC-specific antibodies in combination removed the upper band (**a**) and further reduced band (**b**), compared to the NLRP-3 antibody alone (lane 4). The IgG antibody control did not affect the shifted bands (lane 5) (1 of 3 representative EMSAs). Data is presented as means ± SEMs and analyzed by two-tailed *t*-test. **P < 0.01, ***P < 0.001.

ASC and NLRP-3 were recently identified as transcriptional repressors of *MMP7*; a protease responsible for non-canonical processing of pro-IL-1β in hosts lacking a functional inflammasome^20^. The over-activation of SP/NK1R in *Nlrp3*^−/−^ mice suggested that a similar mechanism might regulate SP/NK1R expression. To address this question, we transfected bladder epithelial cells *in vitro* with *ASC-* or *NLRP3-*specific siRNAs (17 hours) and confirmed the inhibition of ASC and NLRP3 expression by confocal imaging and western blot analysis (*P* < 0.001 for *NLRP3-* and *ASC-*siRNA transfected cells, Fig. 6a and Supplementary Fig. S8). The transfected cells were then infected with CY-17 (MOI = 0.05, 4 hours) and changes in SP/NK1R expression were analyzed (Fig. 6b–d).

NK1R and SP staining increased by about 70% in cells transfected with *ASC-* or *NLRP3*-specific siRNA (Fig. 6b–d and Supplementary Fig. S8). The effect was further enhanced by infection, compared to the scrambled siRNA control or non-transfected cells (*P* < 0.01 for *NLRP3-* or *ASC-*siRNA transfected cells, respectively), (Fig. 6b–d). To exclude that this effect was secondary to ASC and NLRP-3 dependent IL-1β activation, the transfected cells were treated with the IL-1R antagonist Anakinra. An IL-1β independent increase in NK1R was detected (Supplementary Fig. S8).

The results suggested that NLRP-3 and ASC may bind to *TACR1* promoter DNA and act as repressors of *NK1R* expression (see model in Fig. 6f). This question was addressed, using a 214 base pair *TACR1* promoter fragment as a template in an Electro Mobility Shift Assay (EMSA, Fig. 6g). Lysates from uninfected HTB-9 cells created a triple band shift (a, b and c in Fig. 5h) and ASC and NLRP3 were identified as potential binding partners. Specific NLRP-3 antibodies inhibited the formation of band a and attenuated band b. Anti-ASC antibodies reduced band a and the two antibodies in combination inhibited bands a and b, consistent with the formation of an ASC and NLRP-3 complex on promoter DNA.

### SP response in acute cystitis patients

**Figure 7:**
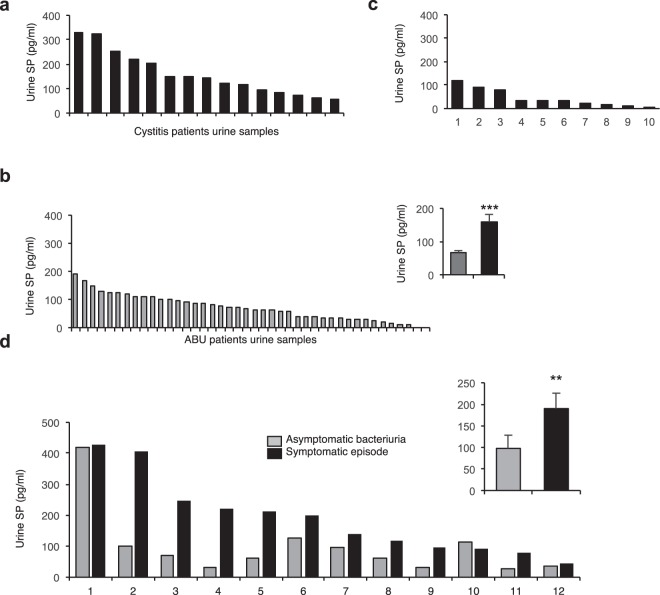
Urine SP levels in patients with acute cystitis compared to asymptomatic bacteriuria. Urine SP concentrations were quantified by ELISA in patients diagnosed with acute cystitis compared to healthy controls and patients with long-term ABU. (**a**) Patients with acute cystitis (*n* = 15). (**b**) Patients with long-term ABU (*n* = 40). Histogram (inset) shows elevated mean SP concentrations in patients with acute cystitis compared to ABU (Mann-Whitney U-test). (**c**) Healthy controls (n = 10). (**d**) Quantification of SP levels in paired urine samples obtained during asymptomatic carriage of *E. coli* 83972 or symptomatic episodes from the lower urinary tract caused by other bacterial strains^24^ (*n* = 12 patients, 24 samples, Wilcoxon signed rank test). The red lines indicate the detection limit of the ELISA. The data is presented as means + SEMs. **P < 0.01.

Relevance of these findings to acute cystitis was supported by quantification of SP levels in patient urine. Patients with acute cystitis had significantly higher urine SP levels at the time of diagnosis (*n* = 15 samples from 13 patients), than patients with asymptomatic bacteriuria (ABU, *n* = 42 samples from 20 patients, *P* < 0.001, Fig. 7a,b)^24^. Low SP levels were detected in urine samples from healthy controls (Fig. 7c).

In a second analysis, we compared paired urine samples obtained from patients with asymptomatic carriage of *E. coli* 83972 who experienced symptomatic flares from the lower urinary tract induced by super-infection with a different strain. All but one of the patients had higher urine SP levels at the time of symptoms than during ABU (*n* = 24 samples from 12 patients, 191 pg/ml vs. 109 pg/ml by ELISA, *P* = 0.004, Fig. 7d). The results suggest that acute cystitis is accompanied by a SP response in the bladder mucosa.

## Discussion

Infections threaten the integrity of mucosal surfaces, which retaliate, with the help of an intricate and a tightly controlled anti-microbial defense. Epithelial cells are essential for the mucosal barrier function and, when activated, they recruit a range of resident and circulating cells, to execute the defense. Mucosal surfaces are also richly innervated^25,26^ and it has been proposed that the mucosal immune system is closely controlled by the nervous system^27,28^. Here we show that the mucosal immune response is regulated by direct bacterial effects on nerve cells and epithelial cells, through the activation of neuropeptides and neuropeptide receptors. The example is acute cystitis, a bacterial infection of the urinary bladder characterized by pain at voiding, urgency and frequency of urination. We show that the pathogenesis of acute cystitis involves infected nerve cells and that epithelial cells resemble nerve cells, in that they express neuropeptide receptors and secrete neuropeptides in response to infection. The results suggest that a concerted action of these two cell types may contribute significantly to pain at the site of infection and increased afferent and efferent CNS activity, which accompany mucosal infections. We also propose that these receptors may be targeted therapeutically, to alleviate symptoms associated with acute infection.

**Figure 8:**
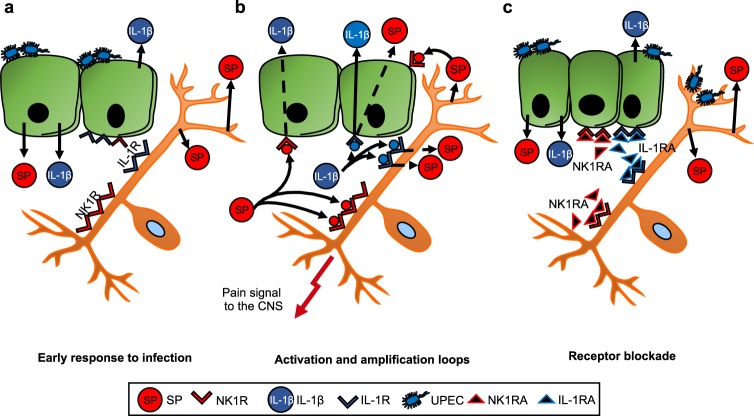
Model of the neuroepithelial response to *E. coli* infection – “the IL-1 - neurokinin loop”. (**a**) *Early response*: Bacteria activate SP/NK1R expression in bladder epithelial cells, which are in parallel activated to produce IL-1R and to secrete IL-1β, creating an inflammatory response. (**b**) *Activation and amplification loops:* SP stimulates NK1R activation in nerve cells and activates IL-1β secretion in bladder epithelial cells, as shown by adding SP to uninfected cells. IL-1β then activates SP/NK1R expression in both nerve- and epithelial cells, which express IL-1R. (**c**) *Receptor blockade:* By treating the cells with IL-1RA or NK1R antagonists (NK1RA) both the pain signal and the inflammatory signal are inhibited (Supplementary Fig. S9). Drawings were modified from biodraw pictures (© motifolio.com).

The bladder epithelium has been proposed to share key features with sensory neurons^29^, including the ability to express neuropeptides and neuropeptide receptors^30,31^. As a result, the epithelium may participate in the regulation of pain and influence the micturition reflex, either by a direct myogenic detrusor effect^32^ or by causing increased afferent activity^33^. This hypothesis is supported by the results of the present study. In addition, ligand release by each infected cell type was shown to trigger an amplification loop for co-activation of both cell types. We speculate that the symptoms of acute cystitis might be caused by the combined activation of the epithelial barrier and mucosal nerve cells (see model, Fig. 8). Triggered directly by infection, this response may increase afferent activity via C fibers, extending to the spinal cord and central nervous system, resulting in nociception as well as increased efferent activity, activating the lamina muscularis^34,35^. Additional cells in the lamina propria might play a role in this loop as well, including eosinophils and mast cells, which play an important role in acute cystitis and are known to produce and release SP in mice models and patients with interstitial cystitis/bladder pain syndrome^36–39^.

SP/NK1R signaling has also been linked to pro-inflammatory signaling in mast cells and macrophages, and in astrocytes, IL-1β induces NK1R expression^8,40,41^, suggesting that neurocrine and innate immune responses may converge. The genetic control of the NK1R/SP response was investigated here using mice carrying single gene deletions of *Tlr4*, *Il1b*, *Asc* and *Nlrp3*. Two patterns were observed. *Tlr4*^−/−^ and *Il1b*^−/−^ mice showed an attenuated phenotype, with markedly reduced background NK1R/SP expression and little or no response to infection. Consistent with the roles of *Tlr4*, *Il1b* as regulators of innate immunity, the mice were also unresponsive to inflammation and tissue damage and thus protected from disease. In *Asc*^−/−^ and *Nlrp3*^−/−^ mice, in contrast, NK1R and SP responses were markedly increased, as was tissue pathology. Interestingly, this disease phenotype was controlled by a new regulatory node, involving ASC and NLRP-3 as transcriptional repressors of *Tacr1*. The same mechanism has recently been shown to control pro-IL-1β processing, suggesting a common mechanism of transcriptional regulation affecting NK1R expression and IL-1β processing. NF-κB family genes were also regulated during bladder infection in *Nlrp3*^−/−^ mice and *Nfkb* expression was suppressed by SR140333 treatment of infected mice, supporting previous studies indicating that NF-κB might be involved as an upstream regulator of SP/NK1R and pro-inflammatory signaling^8,42^.

The predicted outcome of SP binding to NK1R is pain, as shown in numerous studies^43^ and a link between the neuropeptide response and lower urinary tract symptoms has previously been documented in models of pelvic pain, interstitial cystitis and UTI where an increase in SP-reactive varicose nerve fibers has been observed within the lamina propria, additionally, Duell *et al*. detected an increase in *Tac1* gene expression after bladder infection in mice^44,45^. In addition, elevated urine SP concentrations were detected in patients with interstitial cystitis and in UTI patients with pyuria^46–49^. Consistent with these studies, we found elevated SP levels in patients with acute cystitis compared to patients with ABU and healthy controls. Furthermore, in a longitudinal study of ABU, a pair-wise, intra-individual comparison of SP levels revealed a difference between asymptomatic and symptomatic episodes in individual patients, suggesting that neuropeptides may serve as biomarkers of mucosal involvement in this patient group. Consistent with pain from the urinary bladder area, we detected elevated urine SP levels and a loss of locomotion, lack of rearing and grooming behavior in infected C57BL/6 mice with bladder pathology.

Anti-inflammatory agents are emerging as a novel therapeutic approach in acute cystitis, suppressing the symptoms while the host clears the infection. We have previously shown that inhibition of IL-1β by Anakinra treatment is efficient in the murine acute cystitis model^20^. In this study, we further suggest that NK1R inhibition might constitute an interesting alternative approach to prevent inflammation and pathology. Several studies have documented the therapeutic efficacy of different NK1R antagonists in rodent models of nociception^17,50^, and in patients with irritable bowel syndrome, pain and anxiety was reduced by chronic NK1R antagonist treatment^51^. In addition, NK1R antagonist treatment has proved effective in clinical studies of overactive bladder syndrome, characterized by urgency and frequency, symptoms shared with acute cystitis^52–54^. In clinical trials investigating analgesic effects, these drugs have often failed, however^55^. Our findings suggest that the use of NK1R antagonist therapy should be explored in patients with acute cystitis or recurrent UTIs, where antibiotic resistance is creating an urgent need for novel therapeutic alternatives.

## Methods

### Cellular assays

#### Bacterial strains

The cystitis isolates including CY-17 and ABU isolates were isolated during a prospective study of childhood UTI in Gothenburg, Sweden^56,57^ *E. coli* CFT073 (O6:K2:H1)^58^ and *E. coli* 83972 (OR:K5:H-)^59^ were used as controls. Bacteria were cultured on tryptic soy agar plates (TSA, 16 h, 37 °C), harvested in phosphate buffered saline (PBS, pH 7.2) and diluted to appropriate concentration for infection.

#### Cell culture

Grade II human bladder epithelial cells HTB-9 (ATCC, 5637), kidney epithelial cells A-498 (ATCC, HTB-44), and DLD-1 colon epithelial cells (ATCC, CCL-221) were cultured in RPMI-1640 and human neuroblastoma cells (ATCC, SH-SY5Y) were cultured in DMEM/F12 supplemented with sodium pyruvate, non-essential amino acids and 10% heat inactivated FBS and incubated at 37 °C with 5% CO_2_.

#### *In vitro* infection

HTB9, A498 and DLD1 cells were grown on 8-well glass chamber slides (6 × 10^4^ cells/well) or in 6 well plates (6 × 10^5^ cells/well) overnight in media supplemented with 10% FBS. SH-SY5Y cells were differentiated in 8-well chamber slides (2 × 10^4^ cells/well) or 6 well plates (1.5 × 10^5^ cells/well) using 1% Retinoic acid and serum starvation for 7 days^60^. Cells were washed with PBS and serum free media were added prior to infection with appropriately diluted bacteria in PBS (MOI = 0.05) and incubated for 4 hours at 37 °C with 5% CO_2_.

#### NK1R and IL-1*β* inhibition assays

Cultured cells were pre-treated with NK1R antagonists (5–500 ng/ml) (SR140333, CP-99994 or L-703 606) or IL-1RA (500 ng/ml) (Kineret, Sobi) 30 minutes before infection.

#### NLRP3 and ASC siRNA transfection

HTB-9 cells were transfected with PYCARD/ASC or NLRP3 specific siRNAs (0.09 μM, Flexi-Tube GeneSolution, #GS29108 and #GS114548, Qiagen) or with AllStars Negative Control siRNA (#SI03650318, Qiagen) using the HiPerFect Transfection Reagent (#301705, Qiagen) for 17 hours, then infected.

#### Immunostaining of nerve cells and epithelial cells, *in vitro*

Cells were stained using anti-NK1R. anti-substance P, anti-NLRP3, anti-ASC anti-IL-1β as well as antibodies to nerve cell markers anti-βIII tubulin and anti-NeuN (5% FBC overnight at 4 °C) followed by appropriatly Alexa fluor conjugated secondary antibodies (Molecular Probes) (5% FBS and 0.025% Triton X-100, 1 hour at room temperature), then counterstained with DRAQ5 (Abcam) and examined by laser scanning confocal microscope (Carl Zeiss) and quantified by ImageJ. Antibody controls included primary antibody absorption with specific antigens to NK1R or SP before staining (5:1 ratio, 16 hours at 4 °C), and secondary antibody controls (Supplementary Fig. S10).

#### Western blotting

Cells were lysed with NP-40 lysis buffer or by NE-PER Nuclear and Cytoplasmic Extraction Reagents (Thermo Fisher Scientific) supplemented with protease and phosphatase inhibitors (Roche Diagnostics). 7 µg of proteins were run on SDS-PAGE (4–12% Bis-Tris gels, Invitrogen) and blotted onto PVDF membranes (GE Healthcare), blocked (5% NFDM) and stained using anti-NK1R, anti-Substance-P, anti NLRP-3 or anti-ASC primary antibodies. β-actin or GAPDH served as the loading control. Bands were imaged using ECL plus detection reagent (GE Health Care) and were quantified by ImageJ.

#### ELISA

SP and IL-1β in filtered supernatants from uninfected and infected cells were measured by Substance P parameter kit (R&D systems) or IL-1β DuoSet (R&D systems).

#### Electromobility shift assay (EMSA)

A 214 DNA fragment from the *TACR1* promoter was used as probe and stained with GelGreen (Biotium). Each reaction contained 3–5 μg of DNA probe and 5 μg of cell extracts from uninfected HTB-9 cells in binding buffer (100 mMTris, 500 mM NaCl and 10 mM DTT, pH 7). For the band shift competition assay, 1 μg of anti-ASC or anti-NLRP3 antibodies were used separately and together. Binding reactions were incubated at 15 °C for 30 min, loaded onto a 6% non-denaturing, non-reducing polyacrylamide gel and ran in a 50 mM Tris (pH 7), 0.38 M glycine, and 2 mM EDTA buffer at 125 V for 2 hours. Mouse IgG2A isotype control was used as negative control antibody. Gels were imaged using the Bio-RAD ChemiDoc system.

### PMN and PBMC isolation and flow cytometry

PMNs and PBMCs were isolated from healthy controls according to the protocol from Olsson *et al*.^61^. Isolated PBMCs and PMNs were stimulated with filtered supernatants from infected or uninfected HTB-9 cells (1 h at 37 °C) before staining using anti-NK1R primary antibodies (PBS, 45 min RT) and alexa-488 conjugated secondary antibodies (PBS, 30 min RT). The stained cells were analyzed using Accuri C6 (BD biosciences).

### Mice

Female C57BL/6 mice or *Tlr4*^−/−^*, Il1b*^−/−^ ^62,63^, *Nlrp3*^−/−^, *Asc*^−/−^ ^64^ mice were used for experiments at 9–15 weeks of age. *Nlrp3*^−/−^ and *Asc*^−/−^ mice were from Jürg Tschopp’s laboratory, Department of Biochemistry, University of Lausanne and Institute for Arthritis Research (aIAR). *Il1b*^−/−^ mice were generated by the Iwakura lab, Laboratory Animal Research Center, Institute of Medical Science, University of Tokyo. *Tlr4*^−/−^ mice were generated in the BIKEN animal facilities, Osaka, Japan. Mice were bred and housed in the specific pathogen-free MIG animal facilities (Lund, Sweden) with free access to food and water. For number of mice used, see respective figure legend.

#### Experimental acute cystitis

Mice were intravesically infected with CY-17 under Isofluorane anesthesia (10^8^ CFU in 0.1 ml), through a soft polyethylene catheter. Pain behavior (lack of rearing, lack of locomotion and grooming behavior) was recorded for each mouse for 3 minutes in a clear cage at 24 hours and 7 days, modified from Ruddick *et al*.^65^. Bladders were aseptically removed at sacrifice and documented by photography for gross pathology analysis, before being embedded in O.C.T compound for H&E and IHC analysis as previously described^20^, scoring was non-blinded and performed by two independent researchers. Urine samples were obtained before infection and at regular times after infection. Bacterial burden was quantitatively cultured and urine neutrophils were quantified in a hemocytometer. Urine SP was quantified by Substance P parameter kit (R&D systems).

#### NK1R and IL-1RA therapy

SR140333 was injected intraperitonally (i.p.) in *Nlrp3*^−/−^ (1 mg/kg), either 1 hour prior to infection or 30 minutes after infection with CY-17 for 24 hours. C57BL/6WT mice were treated with the post-infection regime (1 mg/kg) 24 hours or 7 days. IL-1RA was injected (1 mg/kg, i.p.) in C57BL/6WT mice 1 hour prior to CY-17 infection for 24 hours.

#### Immunohistochemistry

7-µm-thick cryosections were mounted on positively charged microscope slides and stained as previously described using anti-NK1R, anti-substance P, anti-βIII tubulin antibodies, anti-neutrophil and anti-macrophage antibodies^20^. Sections were imaged by laser scanning confocal microscopy or by fluorescence microscopy. Staining controls included antibody absorption and secondary antibody controls (Supplementary Fig. S10).

#### mRNA isolation

Total RNA was extracted from murine bladders in RLT buffer with added β-Mercaptoethanol (1%) after disruption in a tissue homogenizer (TissueLyser LT, Qiagen) using Precellys® Lysing kits (Bertin Technologies), with the RNeasy® Mini Kit (Qiagen).

#### Global gene expression in infected bladders

Total bladder RNA was amplified using GeneChip 3´IVT Express Kit, hybridized onto Mouse Genome 430 PM array strips (16 hours at 45 °C), washed, stained and scanned using the Geneatlas system (Affymetrix). Data was normalized using Robust Multi Average implemented in the Partek Express Software (Partek). Significantly altered genes were sorted by relative expression (2-way ANOVA model using Method of Moments, *P*-values < 0.05 and absolute fold change >1.41) and analysed using Ingenuity Pathway Analysis software (Ingenuity Systems, Qiagen) and ToppGene^66^. Heat-maps were constructed using Gitools 2.1.1 software.

#### Quantitative RT-PCR

Quantitative RT-PCR was performed as previously described using primers pairs against *Mus Musculus Tacr1*, *Ppt-A, Cxcl2 and Il1b* per the MIQE guidelines on a Rotor Gene Q (Qiagen) (Supplementary Table S3)^20,67^. qRT-PCR reactions were run in technical duplicates and gene expression was analyzed based on ***ΔΔ***CT comparison to *Mus Musculus Gapdh*.

### Clinical urine samples

Urine samples from adult patients with community acquired acute cystitis were collected at two primary health care units in Lund, Sweden^20^. A diagnosis of acute cystitis was based on a urine dipstick analysis positive for bacteria and lower urinary tract symptoms (dysuria, suprapubic pain and no fever). Urine samples from patients with asymptomatic bacteriuria were obtained from a previous study^24^ from patients who carried *E. coli* 83972 asymptomatically or from the control arm of the study (Placebo control or after spontaneous clearance of *E. coli* 83972). Urine SP levels were quantified by ELISA using the Human Substance-P ELISA kit (ab133029, Abcam).

#### Ethical statement

Experiments were approved by the Malmö/Lund Animal Experimental Ethics Committee at the Lund District Court, Sweden (numbers M104-10 and M44-13). All animal care and protocols were governed by the European Parlement and Council Directive (2016/63, EU) The Swedish Animal Welfare Act (Djurskyddslagen 1988:534), the Swedish Welfare Ordinance (Djurskyddsförordningen 1988:539) and Institutional Animal Care and Use Committee (IACUC) Guidelines. All the experiments were reported per the ARRIVE guidelines. The studies of human UTI were approved by the Ethics Committee of the medical faculty, Lund University, Sweden (LU106-02, LU236-99, Dnr 298/2006; 463/2010 and Clinical Trial Registration RTP-A2003, International Committee of Medical Journal Editors, www.clinicaltrials.gov), Patients gave their informed written consent and all experiments were performed in accordance with the relevant guidelines and regulations.

### Statistics

Unpaired *t*-tests and one-way ANOVA (Bonferroni for Post-Hoc analysis) were used for data determined to follow a normal distribution defined by D’agostino & Pearson normality test. Mann-Whitney U-tests, Wilcoxon signed ranked tests and Kruskal Wallis tests (Dunn’s test for Post-Hoc analysis) were used for non-parametric analyses. Welsh’s *t-*test were used to determine statistics for kinetic responses. *P < 0.05, **P < 0.01 and ***P < 0.001. The data was examined by using Prism (v. 6.0 GraphPad).

## Electronic supplementary material

Supplementary Dataset 1
