# Supplementary material for: Neuroepithelial control of mucosal inflammation in acute cystitis
Source: Sci Rep. 2018 Jul 20;8:11015. doi: 10.1038/s41598-018-28634-0 (PMC6054610; doi:10.1038/s41598-018-28634-0)
Supplement: Supplementary file 1 — Supplementary Dataset 1 [file 41598_2018_28634_MOESM1_ESM.docx]

**Supplementary Figures**

**Neuroepithelial control of mucosal inflammation in acute cystitis**

Daniel S.C. Butler^1^, Ines Ambite^1^, Karoly Nagy^1,2^, Caterina Cafaro^1^, Abdulla Ahmed^1^, Aftab Nadeem^1^, Nina Filenko^1^, Thi Hien Tran^1^, Karl-Erik Andersson^3,4^ Björn Wullt^1^, Manoj Puthia^1^, Catharina Svanborg^1*^

^1^Department of Microbiology, Immunology and Glycobiology, Institute of Laboratory Medicine, Lund University, Lund, S-223 62, Sweden

^2^ Jahn Ferenc (South Pest) Teaching Hospital, 1204 Budapest, Hungary

^3^ Institute for Regenerative Medicine, Wake Forest University School of Medicine, Winston Salem, NC, USA

^4^ Institute of Clinical Medicine, Department of Obstetrics and Gynecology, Aarhus University, Aarhus Denmark)

**Correspondence:** Catharina Svanborg

**Email**: Catharina.Svanborg@med.lu.se **Phone**: +46 709-426549

**Fig S1. NK1R and SP responses to acute cystitis isolate CY-17.**


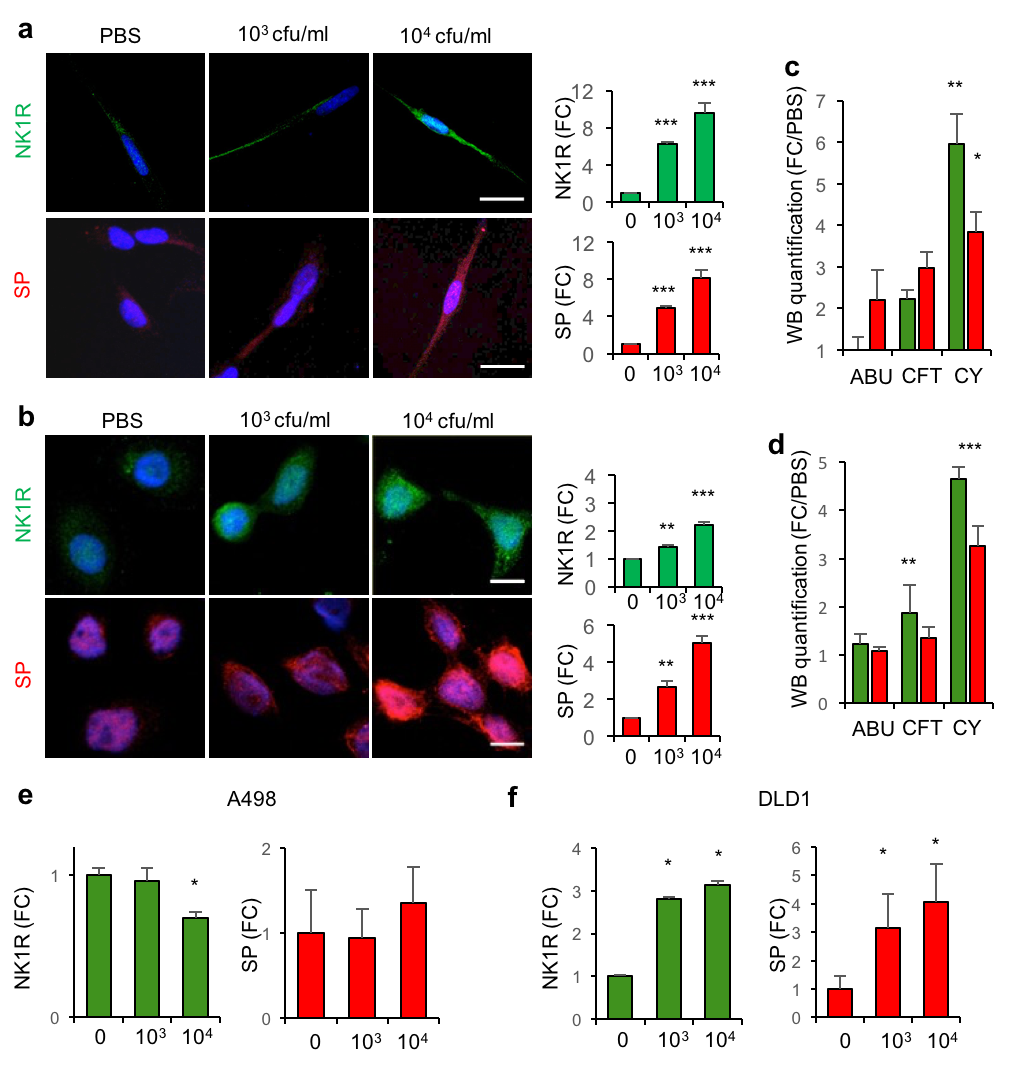


Dose dependent NK1R and SP response to CY-17 infection (10^3^ - 10^4^ CFU/ml, MOI = 0.005 – 0.05, 4 hours) in SH-SY5Y nerve cells and HTB-9 bladder epithelial cells. (**a**) Confocal imaging of the NK1R (green) and SP (red) response in nerve cells with inserted quantification (FC to PBS control) (**b**) Confocal imaging of the NK1R (green) and SP (red) response in bladder epithelial cells. Scale bar = 20 µm. (**c**-**d**) Quantification of Western blots (*n =* 4) from Fig 1d (c) and Fig 1h (d). (**e**) SP and NK1R response in A498 kidney epithelial cells. (**f**) SP and NK1R response in DLD1 colon epithelial cells. Data is presented as means + SD of 3 experiments, 5 cells per condition. * = *P* < 0.05 ** = *P* < 0.01, *** = *P* < 0.001

**Fig S2. Response of nerve cells and bladder epithelial cells to acute cystitis- or and ABU isolates.**

ABU

CY

ABU

CY

SP

NK1R

CY

ABU

ABU

CY

SP

NK1R

**a**

**b**

*P* = 0.012

*P =* 0.026

*P <* 0.001

*P =* 0.027

Nerve cells and bladder epithelial cells were infected with a defined pediatric acute cystitis isolates or pediatric ABU isolates (MOI = 0.05, 4 hours). (A) NK1R (green) and SP (red) staining in SH-SY5Y nerve cells infected with cystitis isolates or ABU isolates. Fold change compared to PBS control. (B) NK1R (green) and SP (red) staining in HTB-9 bladder epithelial cells infected with cystitis isolates or ABU isolates, Fold change compared to PBS control. Mean of three experiments, 50 cells per sample, Mann-Whitney U-test. * = *P* 0.05, ** = *P* < 0.01, *** = *P* < 0.001.

**Fig S3. localization of NK1R and SP.**


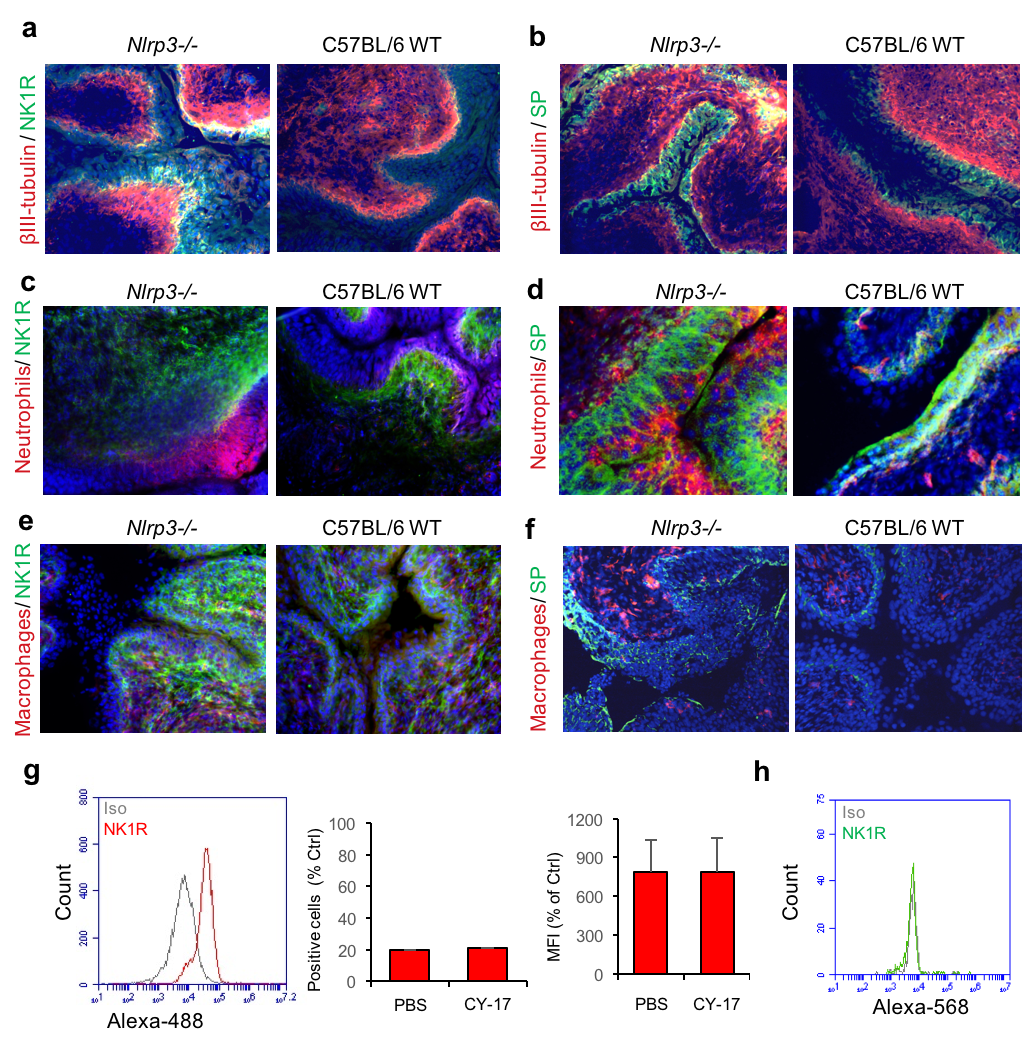


Tissue sections from *Nlrp3^-/-^* and C57BL/6 WT mice (*n =* 4 mice per group) were infected with CY-17 for 7 days and stained for NK1R or SP. The nerve marker βIII tubulin, the neutrophil marker LY6G or the macrophage marker RM0029-11H3, were used. (**a**) Co-localization of NK1R (green) with βIII tubulin (red). (**b**) Co-localization of SP (green) with βIII tubulin (red). (**c**) No co-localization of NK1R (green) with Neutrophils (red). (**d**) No co-localization of SP (green) with Neutrophils (red). (**e**) N No co-localization of K1R (green) with macrophages (red). (**f**) No co-localization of SP (green) with macrophages (red). White arrowheads indicate co-localization. Scale Bars =100 µm. (**g-h**) The lack of PMNs and CD14+ PBMC contribution to this response was confirmed *in vitro* by stimulating isolated PMNs (**g**) or PBMCs (**h**) from healthy controls (*n =* 5) with supernatants from CY-17 infected bladder epithelial cells (1 hour).

**Fig S4. Protected phenotype of infected *Tlr4-/- and Il1b-/- mice.***

**
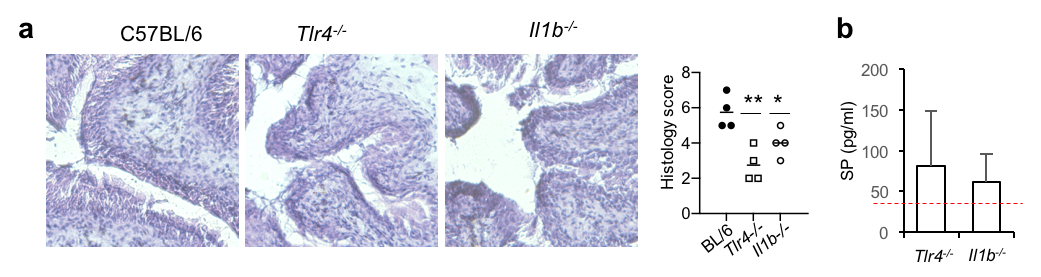
**

(**a**) H&E stained bladder tissue sections from *Tlr4^-/-^ and Il1b^-/-^* mice and tissue pathology score, compared to infected WT mice after CY-17 infection for 7 days (*n =* 4 mice per infection group, Mann-Whitney test). (**b**) Urine SP levels in CY-17 infected *Tlr4^-/-^ and Il1b^-/-^* mice (*n =* 4 mice per infection group compared to uninfected controls).

**Fig S5. Effect of SR140333 treatment in *Nlrp3^-/-^* mice and C57BL/6 WT mice**

**
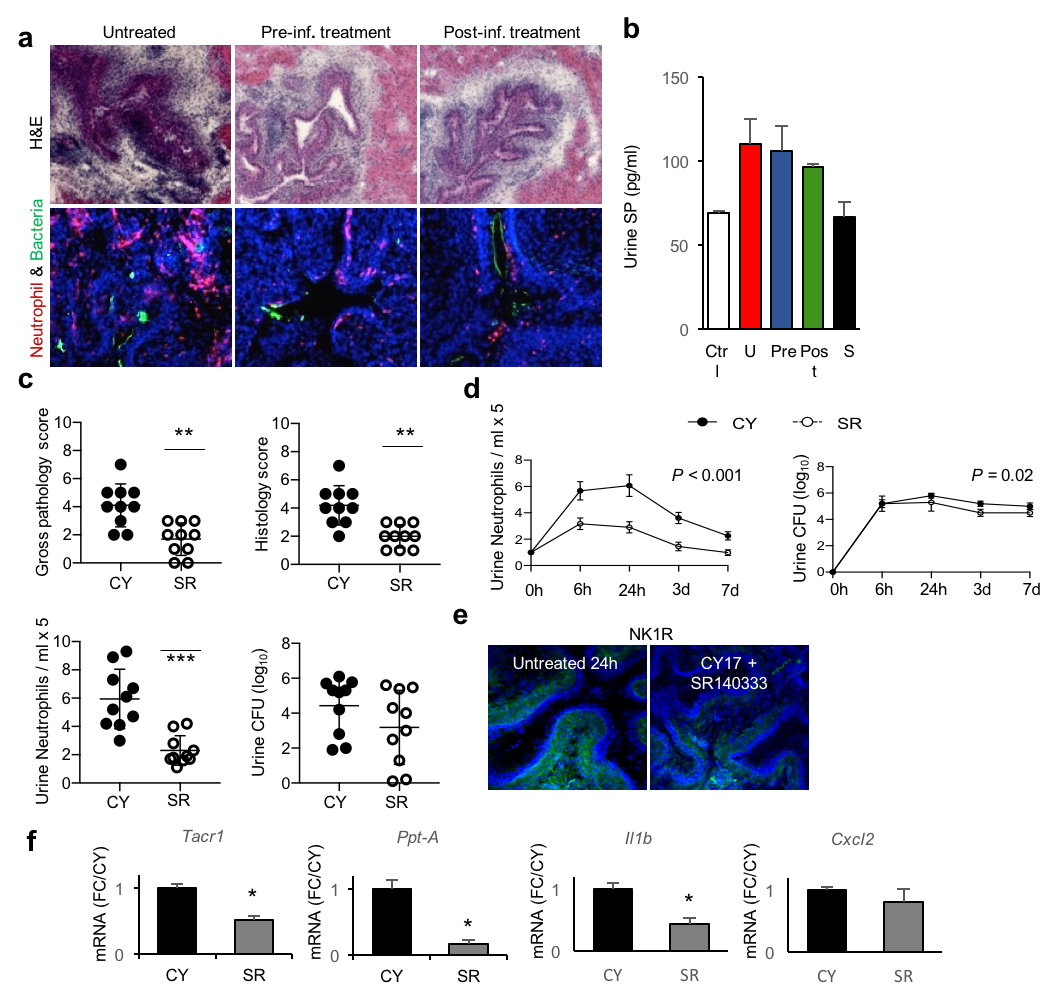
**

(**a**) Evidence of acute cystitis in bladder tissue sections from CY-17 infected *Nlrp3*^-/-^ mice. Rescue after treatment with the NK1R inhibitor SR140333 (H&E staining, *Nlrp3^-/-^* mice, 24 hours after infection, Scale bar = 100 µm). (**b**) SP ELISA from urine of infected *Nlrp3*^-/-^ mice treated with SR140333 or left untreated (24 hours). No change in SP release by SR140333, either by the pre- or post-treatment regime (*n =* 10 mice per group except sham and uninfected where *n =* 4). (**c**) Protective effect in C57BL/6 WT mice, treated with SR140333 (1 mg/kg i.p.1 hour after infection with CY-17), compared to untreated control mice. Decrease in gross pathology score, histology score and urine neutrophil counts. The inhibitor did not change bacterial counts in urine (means ± SEMs, *n =* 10 mice per group, 2 experiments). (**d**) Kinetics of urine neutrophil recruitment and urine bacterial counts in C57BL/6 WT mice infected with CY-17. SR140333 treated C57BL/6 WT mice are compared to untreated, infected WT mice (means + SEMs, *n =* 6 per group, two experiments). (**e**) NK1R staining in C57BL/6 WT mice infected with CY-17 for 24h compared to C57BL/6 WT mice infected with CY-17 and treated with SR140333*.* (**f**) Effect of SR14033 on *Tacr1, Ppt-A, Il1b and Cxcl2* gene expression in C57BL/6 WT mice infected with CY-17 compared to untreated infected controls. Data is presented as means + SEMs, Mann Whitney tests for all except kinetics where AUC analysis with Welch’s *t*-test was performed. * = *P* < 0.05, ** = *P* < 0.01

**Fig S6. NK1R inhibition *in vitro***

**
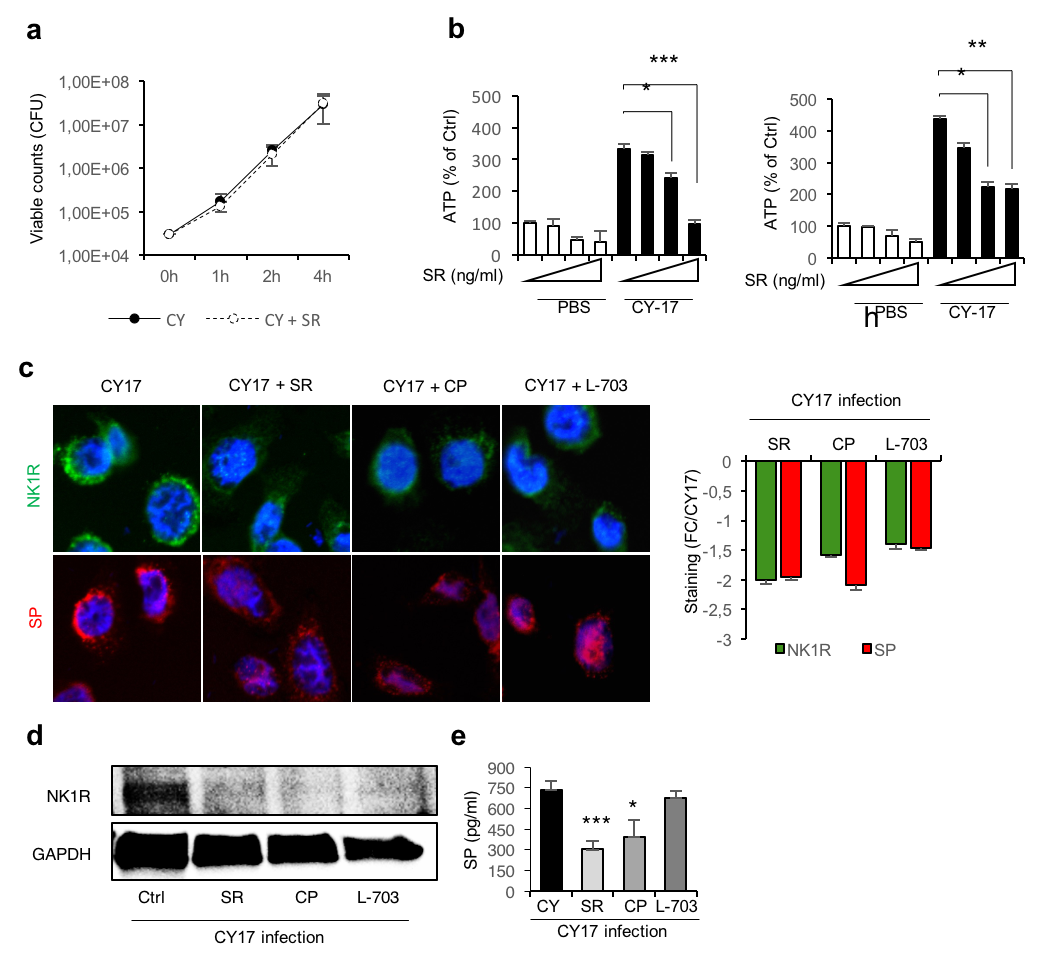
**

**(a)** CY-17 was grown in LB broth with the addition of vehicle (DMSO) or SR140333 to show whether there are bactericidal effects of SR140333. (**b**) downstream effects of SR140333 on ATP release in nerve- and bladder epithelial cells showing dose dependent decrease (5-500 ng/ml) of ATP release in both cell lines. (**c**) Additional NK1R antagonists were investigated in HTB-9 bladder epithelial cells by confocal microscopy (histogram inset shows NK1R and SP staining compared to CY-17 infected cells). (**d**) Western blot analysis confirming (c). (**e**) SP release in bladder epithelial cells confirming (c).

**Fig S7. Genes regulated by NK1R inhibition in infected *Nlrp3^-/-^ mice.***


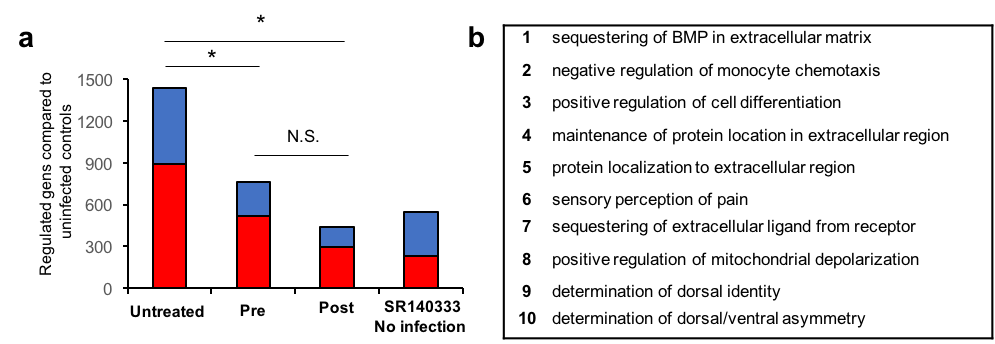


(**a**) Number of regulated genes (up = red, down = blue) in *Nlrp3^-/-^* mice treated with SR140333 compared to infected controls without treatment or uninfected controls (*n =* 2 per group, Chi^2^-test). (**b**) Top 10 biological functions Inhibited by SR140333 treatment post CY-17 infection compared to untreated, infected controls. * = *P* < 0.05.

**Fig S8.** **Inflammasome control of mucosal neurokinin control**


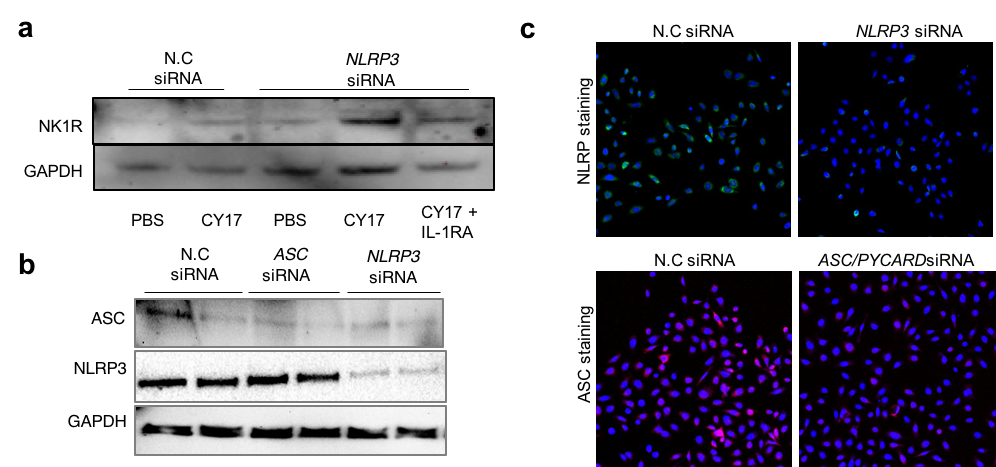


(**a**) As a specificity control to exclude the involvement of IL-1β, cells were pre-treated with IL-1RA (Anakinra, 500 ng/ml, 30 minutes) and the change in NK1R expression was quantified by western blot analysis. (**b**) Suppression of NLRP3 and ASC expression by siRNA treatment (control for Fig 6d). (**c**) Suppression of NLRP3 and ASC expression by siRNA treatment (control for Fig 6a). Data is presented as Means + SEMs, *n =* 3 experiments, one-way ANOVA, Bonferroni or two-tailed *t-*test.

**Fig S9. The IL-1β and SP loop.**

**
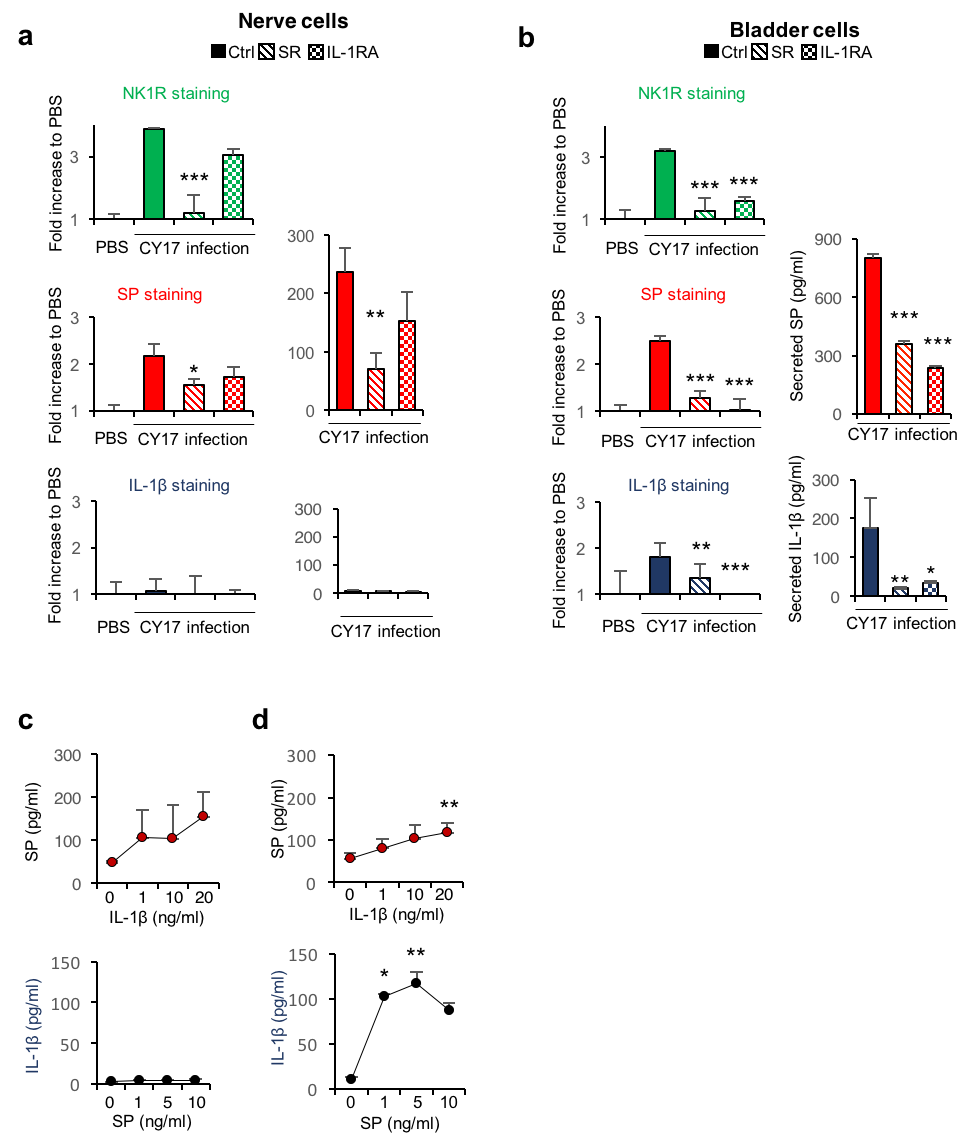
**

To understand the link between SP/NK1R signaling and IL-1β, nerve cells and bladder epithelial cells were pre-treated with SR140333 or IL-1RA (500 ng/ml) 30 minutes prior to CY-17 infection before examining the changes in SP, NK1R and IL-1β expression. (**a**) Pre-treatment with SR104333 reduced NK1R and SP expression in nerve cells. As nerve cells neither produced or secreted IL-1β, there was no significant effect of IL-1RA on SP/NK1R expression (50 cells per condition). (**b**) In bladder cells, NK1R, SP and IL-1β expression were reduced by both SR140333 and IL-1RA (50 cells per condition). (**c**) Nerve cells were treated with exogenous IL-1β (1-20 ng/ml) or recombinant (1-10 ng/ml) for four hours before investigating secreted IL-1β or SP. Exogenous IL-1β triggered SP secretion, but recombinant SP were not able to trigger IL-1β secretion (*n =* 5 samples per condition). (**d**) The effect of exogenous treatment with IL-1β or SP were also performed in bladder epithelial cells. Exogenous IL-1β triggered SP secretion and recombinant SP triggered IL-1β secretion (*n =* 6 samples per condition). Data is presented as means + SEMs from three experiments and analyzed using two tailed *t*-test or Mann-Whitney U-test (c-d), * = 0.05*,* ** = *P <* 0.01*,* *** = *P* < 0.001*,*

**Fig S10. Antibody controls for tissue- and cellular stainings.**

**c**

**b**

**a**


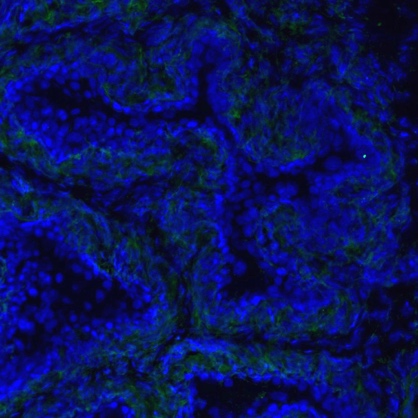

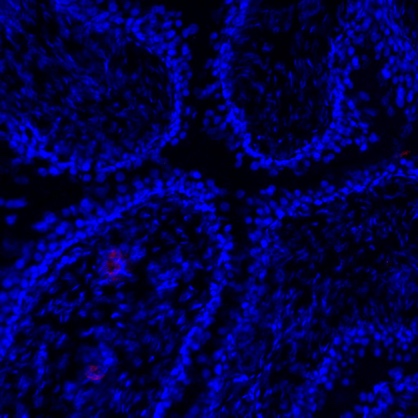


AF 488 Goat anti-mouse

AF 568 Goat anti-rabbit


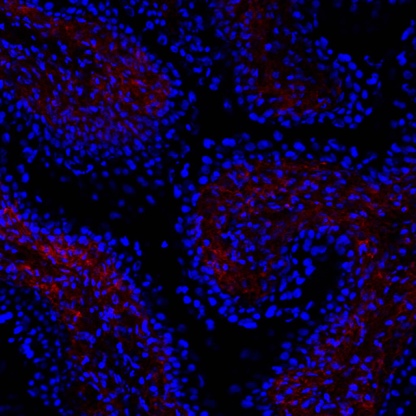


βIII tubulin absorption control


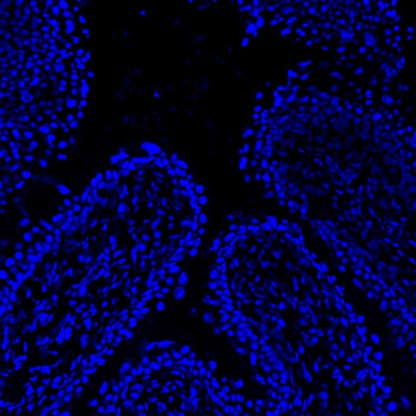


NK1R & SP absorption control


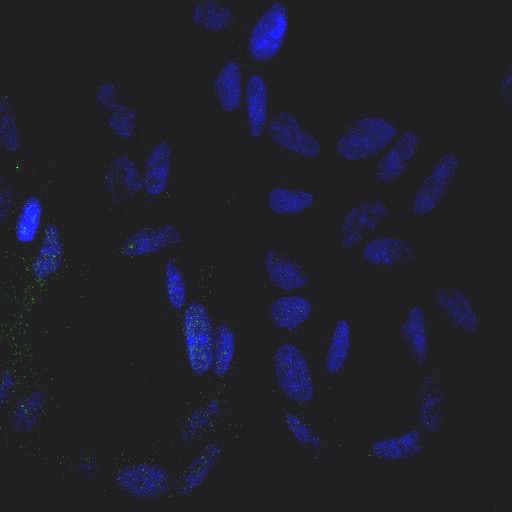


AF 488 Goat anti-mouse


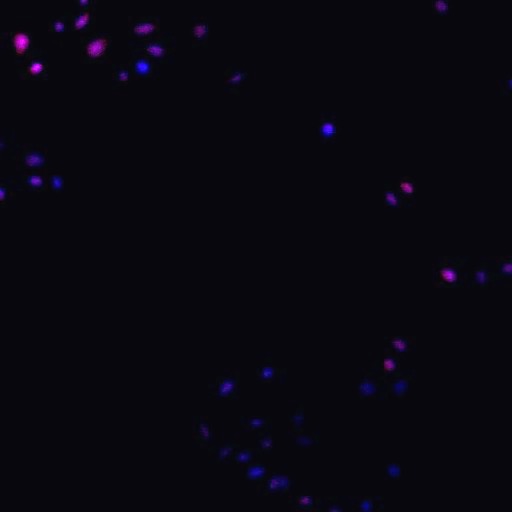


AF 568 Goat anti-rabbit

(**a)** Alexa fluor (AF) 488 (goat anti-mouse) and 568 (goat anti-rabbit) conjugated secondary antibody controls. Nerve cells and bladder epithelial cell were stained. (**b**) AF 488 (goat anti-mouse) and AF 568 (goat anti rabbit) conjugated secondary antibody controls. Tissue sections from uninfected C57BL/6 WT mice were stained. (**c**) Antibody specificity control. Antibodies to SP, NK1R and βIII tubulin were mixed with peptides corresponding to each antigen at a 5:1 ratio.

**Fig S11. Western blots from main figures.**


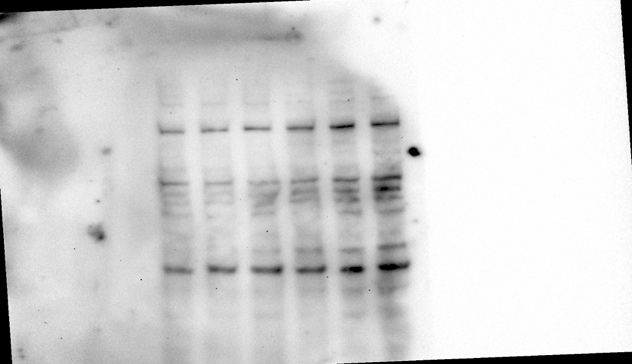

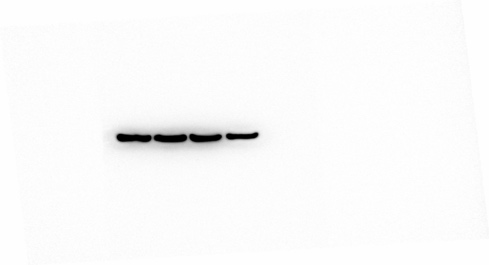


β-actin


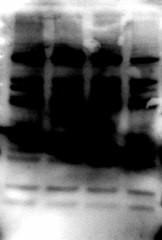


SP


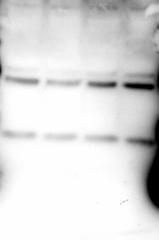


NK1R

40-50

KDa

14 KDa

20 KDa

46 KDa

74 KDa

**a**

74 KDa

100-110

KDa


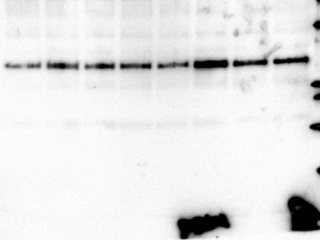

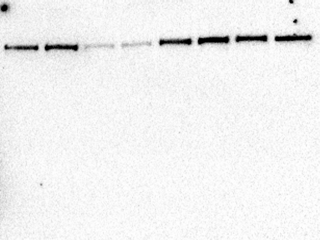

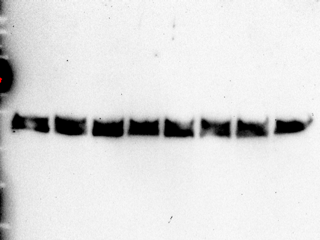

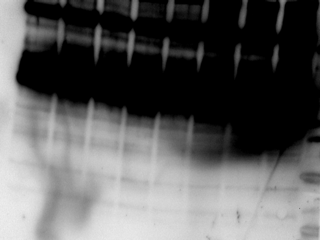


NK1R

NLRP3

ASC

GAPDH

18 KDa

30-40

KDa


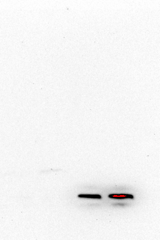

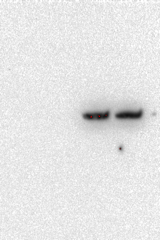

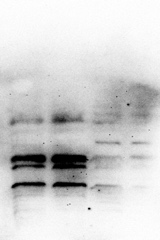


NLRP3

GAPDH

HHH3

15 KDa

30-40

KDa

100-110

KDa

**b**

**d**

**e**


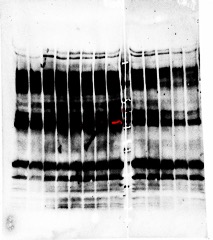


46 KDa

74 KDa


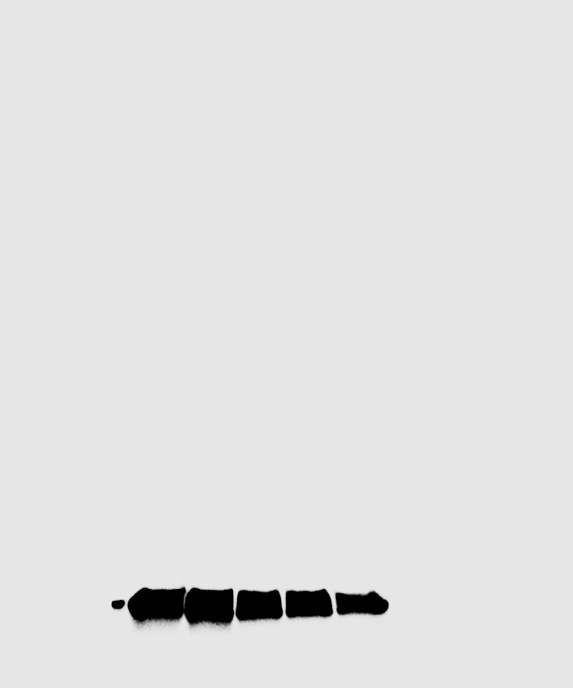


30-40

KDa

NK1R

GAPDH

**c**


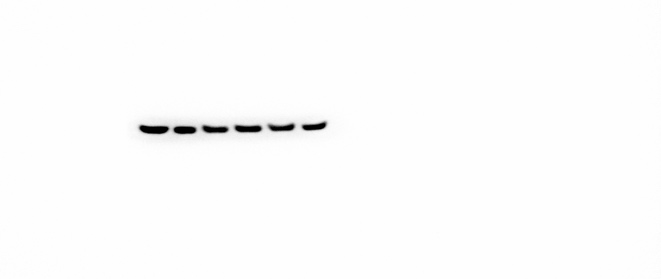

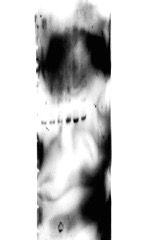


40 -50

KDa

14 KDa

74 KDa

SP

NK1R

β-actin

(**a**) Showing full blots from figure 1d. (**b**) Showing full blots from figure 1h. (**c**) Showing full blots from figure 4h. (**d**) showing full blots from figure 6d and controls in Supplementary Fig S7. (**e**) Blots from Supplementary fig S8.

**Supplementary Table S1. Top biological processes inhibited by SR140333 treatment compared to untreated mice, both 24 hours after infection with CY-17.**

| **GO: Biological Process Name** | **p-value** | **q-value Bonferroni** | **q-value FDR B&H** | **q-value FDR B&Y** | **Hit Count in Query List** | **Hit Count in Genome** | **Hit in Query List** |
| --- | --- | --- | --- | --- | --- | --- | --- |
| Inflammatory response | 1.40E-13 | 5.91E-10 | 5.91E-10 | 5.27E-09 | 32 | 714 | *Il18, Hmgb2, Hmox1, Chi3l1, Ptgs2, Ffar4, Gja1, Hp, Vnn1, Ngfr, Ccr1, C3ar1, Ccr5, Plscr1, Cnr1, Pglyrp1, Ffar2, Casp4, Birc3, Relb, Cxcl2, Cxcl3, Ednrb, Slc11a1, Cd14, Serpine1, Cd44, Tnfaip3, Il1b, Il1rn, Clcf1, Il4r* |
| Regulation of response to stress | 2.27E-12 | 9.58E-09 | 2.23E-09 | 1.99E-08 | 46 | 1585 | *Lgmn, Il18, Nek6, Hmgb2, Wnt4, Hmox1, Ptgs2, Ffar4, Ceacam1, Gja1, Clu, Hp, Vnn1, Nfkbia, Nup210, Ccr1, Plat, C3ar1, Ccr5, Mmp3, Plek, Parpbp, Plscr1, Hspa8, Cnr1, Foxm1, Pglyrp1, Ffar2, Agr2, Casp4, Birc3, Relb, Cxcl2, Ednrb, Ndc1, Cd14, Lmnb1, Clec4d, Mtm1, Serpine1, Cd44, Tnfaip3, Ltf, Il1b, Il1rn, Clcf1* |
| Positive regulation of multicellular organismal process | 6.41E-11 | 2.71E-07 | 4.51E-08 | 4.03E-07 | 44 | 1616 | *Hcar2, Il18, Tnfsf9, Hmgb2, Wnt4, Bcl3, Hmox1, Chi3l1, Lrg1, Ptgs2, Cd84, Gja1, Jun, Clu, Vnn1, Angpt4, Aspm, Ngfr, Ccr1, Plat, C3ar1, Ccr5, Plek, Cnr1, Ffar2, Vtcn1, Casp4, Birc3, Apoc2, Ect2, Cxcl2, Ednrb, Slc11a1, Gpihbp1, Cd14, Mtm1, Serpine1, Tnfaip3, Ltf, Cdc20, Il1b, Tmem100, Clcf1, Il4r* |
| Cytokine production | 8.94E-11 | 3.77E-07 | 4.72E-08 | 4.21E-07 | 28 | 703 | *Pcsk5, Il18, Tnfsf9, Hmgb2, Bcl3, Hmox1, Chi3l1, Ptgs2, Ffar4, Ceacam1, Cd84, Clu, C3ar1, Ccr5, Pglyrp1, Ffar2, Vtcn1, Casp4, Birc3, Relb, Lcp2, Slc11a1, Cd14, Serpine1, Tnfaip3, Ltf, Il1b, Il4r* |
| Apoptotic process | 1.82E-09 | 7.66E-06 | 4.51E-07 | 4.02E-06 | 46 | 1934 | *Lgmn, Hcar2, Il18, Hk2, Nek6, Dapl1, Tnfsf9, Hmgb2, Wnt4, Bcl3, Hmox1, Nr4a1, Pak6, Chi3l1, Ptgs2, Ffar4, Ceacam1, Gja1, Jun, Aldh1a2, Clu, Vnn1, Angpt4, Nfkbia, Bub1b, Ngfr, Ccr5, Plscr1, Hspa8, Cnr1, Pglyrp1, Casp4, Birc3, St6gal1, Ect2, Ednrb, Cd14, Timp1, Serpine1, Cd44, Tnfaip3, Ltf, Ckap2, Il1b, Il1rn, Clcf1* |
| Fever generation | 2.90E-09 | 1.22E-05 | 6.49E-07 | 5.79E-06 | 6 | 15 | *Ptgs2, Ccr5, Cnr1, Ednrb, Il1b, Il1rn* |
| Cell cycle | 3.89E-09 | 1.64E-05 | 7.97E-07 | 7.11E-06 | 43 | 1771 | *Cdkn3, Cenpw, Cenpn, Nek6, Cenpe, Kif4a, Nubp1, Hmmr, Wnt4, Nr4a1, Pak6, Ptgs2, Jun, Ncapg, Nup210, Bub1b, Aspm, Sgo2, Cdt1, Dna2, Hspa8, Kif11, Knl1, Kif22, Foxm1, Kif23, Rbl1, Birc3, Gas7, Ect2, Kif20a, Ndc1, Lmnb1, Fanci, Racgap1, Avpi1, Tnfaip3, Ckap2, Cdc20, Il1b, Rspo1, Tmprss11a, Bora* |
| I-kappaB kinase/NF-kappaB signaling | 2.78E-07 | 1.17E-03 | 2.22E-05 | 1.98E-04 | 14 | 271 | *Il18, Nek6, Bcl3, Hmox1, Ptgs2, Gja1, Nfkbia, Birc3, Relb, Ect2, Cd14, Tnfaip3, Ltf, Il1b* |
| Adaptive immune response | 2.88E-07 | 1.21E-03 | 2.25E-05 | 2.01E-04 | 17 | 406 | *Il18, Bcl3, Ceacam1, Cd84, Clu, C3ar1, Hspa8, Lilrb4, Vtcn1, Relb, Slc11a1, Clec4d, Cd44, Tnfaip3, Il1b, Clcf1, Il4r* |

**Supplementary Table S2. Antibodies used for immunocytochemistry (ICC), Western blot (WB), Immunohistochemistry (IHC) and flow cytometry (FACS).**

| **Antibodies** | **ICC** | **WB** | **IHC** | **FACS** |
| --- | --- | --- | --- | --- |
| Mouse anti-NK1R  sc-514453, Santa Cruz | 1:50 | 1:100 | 1:50 | - |
| Rabbit anti-NK1R  sc-15323 , Santa Cruz | - | - | 1:50 | 1:50 |
| Rabbit anti-Substance P  ORB11399, Biorbyt | 1:200 | 1:500 | 1:100 | - |
| Mouse anti-NLRP-3  AG-20B-0014-C100, AdipoGene | 1:200 | 1:1000 | - | - |
| Rabbit anti-ASC  sc-22514, Santa Cruz | 1:50 | 1:100 | - | - |
| Rabbit anti-Il1b  ab9722, Abcam | 1:200 | - | - | - |
| Mouse anti-bIII tubulin  MAB1195, R&D system | 1:400 | - | 1:400 | - |
| Rabbit anti-NeuN  mabn140, Millipore | 1:200 | - | - | - |
| Rat anti-neutrophil  ab2557, Abcam | - | - | 1:200 | - |
| Rat anti-macrophage  sc-101447, Santa cruz | - | - | 1:50 | - |
| Mouse ani-b-actin  Sigma-Aldrich, A1978 | - | 1:4000 | - | - |
| FITC labeled Mouse anti-CD14  345785, BD | - | - | - | 1:100 |
| HRP-linked GAPDH  sc-25778, Santa Cruz | - | 1:4000 | - | - |
| Alexa488 labeled Goat anti-rabbit  A-11008, Thermo Fischer | 1:400 | - | 1:200 | 1:100 |
| Alexa568 labeled goat anti-rabbit,  A-11011, Thermo Fischer | - | - | - | 1:100 |
| Alexa568 labeled Goat anti-mouse  A-11004, Thermo Fischer | 1:400 | - | 1:200 | - |
| Alexa568 labeled Goat anti-rat  A-11077, Thermo Fischer | - | - | 1:200 | - |
| HRP-linked Goat anti-rabbit  Cell signaling | - | 1:4000 | - | - |
| HRP-linked goat anti-mouse  Cell Signaling | - | 1:4000 | - | - |

**Supplementary Table S3. Murine Primer sequences used for analysis of *Tacr1, Ppt-1, Cxcl2* and *Il1b* gene expression from whole bladder tissue mRNA in uninfected, CY-17 infected C57BL/6 WT, *Tlr4^-/-^, Il1b^-/-^, Asc^-/-^* and *Nlrp3^-/-^* mice, as well as SR140333 treated *Nlrp3* and C57BL/6 WT mice (From Figures 2, 3, 4, and Supplementary Fig S5).**

| **Gene** | **Primer sequences** | **Size (bp)** | | **location** |
| --- | --- | --- | --- | --- |
| *Tacr1* | Forward: 5′-CTTGCCTTTTGGAACCGTGTG-3′,  Reverse: 5′-CACTGTCCTCATTCTCTTGTGGG-3′ | 501 | 6 | |
| *Ppt-A* | Forward: 5′-GCCAATGCAGAACTACGAAA-3′  Reverse: 5′-GCTTGGACAGCTCCTTCATC-3′ | 282 | 6 | |
| *Cxcl2* | Quiagen, QT00113253 | - | 5 | |
| *Il1b* | Origene, MP206724 | - | 2 | |

PCR reactions were performed using the following specifications: Thermal cycling, 95°C, 3 min, followed by 40 cycles comprising a denaturation (95°C, 30 s), annealing (59°C, 30 s), and extension (72°C, 30 s) steps, and a final extension at 72°C, for 6 min.
